# The interplay of SARS-CoV-2 evolution and constraints imposed by the structure and functionality of its proteins

**DOI:** 10.1371/journal.pcbi.1009147

**Published:** 2021-07-08

**Authors:** Lukasz Jaroszewski, Mallika Iyer, Arghavan Alisoltani, Mayya Sedova, Adam Godzik

**Affiliations:** 1 Division of Biomedical Sciences, University of California Riverside School of Medicine, Riverside, California, United States of America; 2 Graduate School of Biomedical Sciences, Sanford Burnham Prebys Medical Discovery Institute, La Jolla, California, United States of America; San Raffaele Hospital: IRCCS Ospedale San Raffaele, ITALY

## Abstract

The unprecedented pace of the sequencing of the SARS-CoV-2 virus genomes provides us with unique information about the genetic changes in a single pathogen during ongoing pandemic. By the analysis of close to 200,000 genomes we show that the patterns of the SARS-CoV-2 virus mutations along its genome are closely correlated with the structural and functional features of the encoded proteins. Requirements of foldability of proteins’ 3D structures and the conservation of their key functional regions, such as protein-protein interaction interfaces, are the dominant factors driving evolutionary selection in protein-coding genes. At the same time, avoidance of the host immunity leads to the abundance of mutations in other regions, resulting in high variability of the missense mutation rate along the genome. “Unexplained” peaks and valleys in the mutation rate provide hints on function for yet uncharacterized genomic regions and specific protein structural and functional features they code for. Some of these observations have immediate practical implications for the selection of target regions for PCR-based COVID-19 tests and for evaluating the risk of mutations in epitopes targeted by specific antibodies and vaccine design strategies.

## Author summary

RNA viruses, such as SARS-CoV-2 have high mutation rates and their genomes accumulate mutations at a pace much faster than larger organisms. While a lot of attention is focused on mutations changing the behavior of the virus, making it more or less infectious or virulent, most mutations appear to be neutral. The interplay between different types of natural selection and genetic drift is intensively studied by viral genetics, with many detailed models of viral evolution. Here we show, on the example of the SARS-CoV-2 virus, that the patterns of mutations in viral genomes are tightly coupled with the three-dimensional structure and detailed functional features of the proteins coded by the viral genome. Highly mutated regions of the genome correspond to structural regions that can easily accept amino acid changes, such as disordered regions or protein surfaces, while the reverse is true for regions corresponding to protein cores or functionally important features. While many patterns can be explained by what we already know about SARS-CoV-2 proteins, others provide hints for the still undiscovered functions or still unknown structural features. Taking into account these patterns may be important when we develop tools, such as antibodies, PCR probes, vaccines or drugs, to make sure we target genomic regions that are conserved because of natural negative selection.

## Introduction

We live in the middle of the COVID-19 pandemic caused by the Severe Acute Respiratory Syndrome Coronavirus 2 (SARS-CoV-2). First identified and characterized in early 2020, the virus is mutating and evolving into separate clades with distinct geographical and time distribution (https://www.gisaid.org) [1]. This is typical for RNA viruses and cannot be automatically interpreted as a sign that the disease it is causing is changing [2], but epidemiological [3] and biochemical [4] data indicate that a viral strain with higher infectivity appeared already in March 2020. Recent pandemic flare ups in the United Kingdom [5] and South Africa [6] suggest that even newer super-transmissible strains are emerging. We can track these events in almost real time thanks to a massive effort in sequencing the SARS-CoV-2 genome variants from all over the world, with most of this information available through resources such as GISAID [1] (https://www.gisaid.org). The genomic data on SARS-CoV-2 provides information on the phylodynamics of the COVID-19 pandemic and is also studied for signals of positive selection in the search of changes that the virus may undergo while adapting to its new host (https://observablehq.com/@spond/revised-sars-cov-2-analytics-page).

There is no doubt that the evolution of the SARS-CoV-2 virus, typical for RNA viruses [7], is mostly driven by a combination of genetic drift and negative (purifying) selection that removes non-viable viruses [8]. Genetic drift and negative or purifying selection typically receive less attention than positive selection since it is considered as being less informative. In this manuscript, we explore the possibility that integrating information about patterns of genetic drift and negative genomic selection with that on protein three-dimensional structures would allow us to gain novel insights about the structurally and functionally important regions of SARS-CoV-2 proteins.

Negative or positive selection is typically measured by a rate of synonymous to non-synonymous mutations and thus can be calculated for individual positions or regions of the genome. Such calculations are carried out for individual variants in the SARS-CoV-2 genome (https://observablehq.com/@spond/revised-sars-cov-2-analytics-page). Here we want to focus only on larger trends, using averages of mutation rates over entire proteins, individual domains or some specific functional regions in them. Such observations were made from the beginning of structural biology, when Perutz and his team noticed that mutations rarely happen in the protein core [9]. These early observations were later corroborated for different organisms, classes of proteins, and evolutionary timescales [10]. It is, however, not obvious if this trend would hold for a rapidly mutating pathogen tracked in the timescale of one year, where neutral genetic drift is expected to be a dominant factor and how much it could be used, in reverse to the earlier analyses, to learn about protein structure and function from the analysis of the mutation patterns. Assuming that negative selection is a dominant effect, we focus on the under and over mutated regions, interpreting the difference as a signal of the importance of these regions to a broadly defined viral fitness.

The analysis presented here is also enabled by the rapid pace of structural characterization of the SARS-CoV-2 proteome [11] as by the end of December 2020, there was direct or indirect high-quality structural information for over 60% of the total length of SARS-CoV-2 proteins. Our group has recently developed the Coronavirus3D server [12], available at https://coronavirus3d.org, to integrate information about the three-dimensional structures of SARS-CoV-2 virus proteins from the Protein Data Bank (PDB) [13] resource (http://rcsb.org) with the information on SARS-CoV-2 genomic variations retrieved from GISAID [1]. This integration allows us to track, in almost real-time, the emergence of new trends or patterns in the evolving SARS-CoV-2 genome. The new functionality of variant tracking is now the default first page and the features described in this manuscript are available from the menu on the top of the page as “3D proteome viewer” or directly at https://coronavirus3d.org/#/3dproteomeviewer.

In the first part of the manuscript, we evaluate mutation rate distributions along the genome to gain insights into the types of selection pressure for individual SARS-CoV-2 proteins as well as for their functional domains and sites. In the second part we analyze the mutation pattern of known antibody epitopes and regions used for COVID-19 diagnostic tests, showing that the continuous evolution of the SARS-CoV-2 virus can also affect the medical and public health aspects of the COVID-19 pandemic and that structural information on viral proteins is useful in our efforts to control it.

## Results

Coronaviruses have a unique RNA copy-proofing mechanism [14] and, as a result, have a lower mutation rate than other RNA viruses. Despite this, over 70% of the positions (21,124 out of 29,880) along the SARS-CoV-2 genome have been mutated at least once, as can be seen by the analysis of over 192,030 high-coverage genomes sequenced as of Dec 3^rd^, 2020 on the GISAID website (https://www.gisaid.org/) (see the Methods section for the details of the protocol used to select these genomes). The distribution of mutations along the SARS-CoV-2 genome has been discussed in many papers [15,16]; here we search for new observations that could be made by mapping this information onto the structures of the proteins encoded by the genome.

### Distribution of mutations along SARS-CoV-2 genome and in its proteins

**Fig 1:**
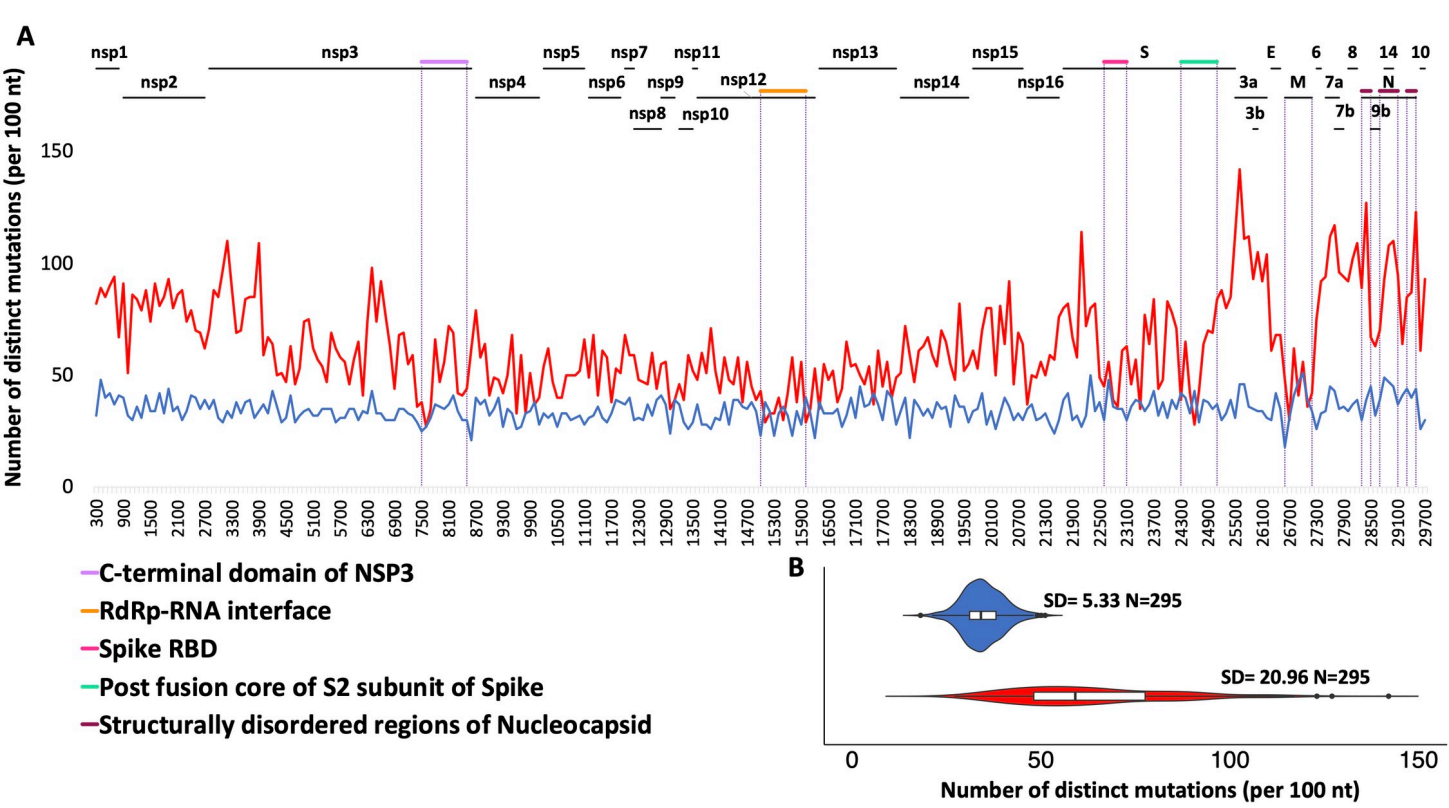
Distribution of SARS-CoV-2 mutations based on multiple sequence alignment of 192,030 high-coverage genomes. **A).** Rate of missense (red) and synonymous (blue) mutations in windows of 100 nt. **B)** Violin plots highlight the differences in the distributions of missense and synonymous mutations in windows of 100 nt. SD; Standard Deviation.

In line with earlier observations [16], the largest proportion of mutations observed in SARS-CoV-2 genomes were missense mutations (61%), followed by synonymous mutations (33%) and a relatively small number of start/stop gains and losses, as well as mutations in untranslated regions (see S1 Fig for more details). When translated to the amino acid sequence, 7811 out of the total of 9926 (79%) amino acids in the SARS-CoV-2 proteome are mutated in at least one genome in the dataset used in this study. We plotted the distribution of missense and synonymous mutations using a moving 100 nt. window along the viral genome (Fig 1A). A cluster of densely mutated regions near the 3’-terminus of the genome begins at the boundary between Orf1ab (coding for non-structural proteins) and Orf2-Orf10 (coding for structural and accessory proteins). Other minima and maxima of the mutation rate can mostly be mapped to the functional parts of the genome as illustrated in Fig 1A. For instance, the region corresponding to the C-terminal domain of nsp3 (violet line in Fig 1A) was found to be significantly less mutated, likely due to its key role in inducing the formation of double-membrane vesicles [17] and the minima in the spike protein to a RBD and postfusion core regions, both critical for the virus entry into the host cell.

As seen in Fig 1B, the variance of the numbers of missense mutations in the 100 nt. windows along the viral genome is about four times higher (20.96 versus 5.33) than the corresponding variance for synonymous mutations (Levene-test p-value = 5.2E-44) confirming that the number of the synonymous mutations fluctuates much less than that of missense mutations.

At the same time, the rates of missense and synonymous mutations along the length of the genome are marginally correlated (Spearman’s rank correlation coefficient Rs = 0.22, p-value = 0.00012) implying that two mechanisms could be coupled- regions under stronger or weaker negative selection on protein level are also slightly more or slightly less frequently mutated overall. A full analysis of this interesting effect is outside the scope of this paper.

Comparing the segment of the genome coding for the non-structural proteins (Orf1ab, corresponding to proteins nsp1-nsp16) to the whole proteome, we see that it is under-mutated in both missense and synonymous mutations (p = 8.01E-75 and 5.72E-6, respectively). We also see that this segment has a lower ratio of missense to synonymous mutations than the segment coding for the structural and accessory proteins (p = 1.85E-12) (see the details of the calculation in the Methods section). This might suggest that negative selection is stronger in the region coding for the essential viral reproduction apparatus, but also that the RNA features of the genome support a lower mutation rate in this region. Therefore, we decided to use the mutation rates in these two regions as two separate background probabilities in the statistical tests applied to individual proteins later in the paper.

### Rates of missense mutations along SARS-CoV-2 genome as a measure of evolutionary pressure

Analysis of the rates of missense mutations in the genomes has a long history in the field of cancer genomics, where cancer mutations have been shown to have clearly non-random distribution when mapped on protein sequences and structures [18]. This effect was interpreted as a signal of positive selection and used to identify cancer driver genes and mutations. It is worth noting that this analogy is not perfect, as in cancer the count of mutations (number of samples) can be directly included in the analysis as they are independent evolutionary events. In contrast, mutations observed in viral genomes are not independent events as individual genomes inherit some mutations from their ancestors. Virus counts are also dependent on the sequencing rates in different regions, with orders of magnitude difference between the industrialized and developing countries. Ideally, the mutation counts should include independent recurrence events. However, existing estimates show a very low frequency of recurrent mutations in SARS-CoV-2 genomes [4,19], on the order of 1% of all positions. They also disagree on the positions of such recurrences as they depend on the details of the phylogenetic tree of all the genomes, which at this point is still not definite [20]. Therefore, we decided to use the approximation of counting each mutation once, without taking into account the virus counts (number of observations) nor the level of recurrence on specific positions since recurrence corrected counts for ~1% of positions would not significantly influence counts averaged over 100nt window, as used here. With rapidly growing number of available genomes this situation may change, but our in-house and literature [4,19] estimates for the datasets as analyzed here support this assumption.

At the same time, as shown in the previous section, variation in the rate of synonymous mutations along the genome is small as compared to missense mutations. Taking into account these observations, we decided to focus entirely on the missense mutations, as they are manifested at the amino acid level and allow us to directly interpret the mutation rates through their effect on proteins, their three-dimensional structures and potentially their functions.

### Some SARS-CoV-2 proteins and domains show significant differences in the rate of mutations

The SARS-CoV-2 genome codes for at least 29 individual proteins, with the product of Orf1ab being further processed into 16 individual non-structural proteins through post-translational processing by the viral proteases 3CLpro and PLpro. The exact count of the proteins coded in the SARS-CoV-2 genome is still disputed, as some of the ORFs code for multiple proteins in alternative reading frames [21]. Many of the SARS-CoV-2 proteins, such as nsp3 or Nucleocapsid phosphoprotein, can be further divided into independently folding regions (domains) with specific functions. In the following analysis, we compared the observed number of missense mutations in a given protein or its domains with their expected number under an appropriate background mutation rate, to identify regions that are significantly over- or under- mutated (see Methods). Because domain assignment is not complete for SARS-CoV-2 proteins, we use information on structurally characterized constructs to define boundaries of structural (and functional) domains or regions, in addition to domains identified by in-depth sequence analysis such as Y1 and CoV-Y domains in nsp3 [22]. Regions located between structurally characterized domains, for instance in nsp3, form another group of indirectly defined regions. The complete list of SARS-CoV-2 proteins and experimentally solved structures/domains within them that are used in the following analysis are listed in S1 and S2 Tables.

While the differences in mutation rates between specific proteins or protein regions could be caused by differences in type of evolutionary pressure between them, they can also be affected by a different “background” mutation rate between different genomic regions. However as discussed earlier the region coding for the non-structural proteins is systematically under-mutated as compared to the region coding for the structural and accessory proteins. Therefore, in this analysis, we used different background frequencies for the different parts of the proteome being analyzed (see Methods). Table 1 presents the results of the significance analysis of the mutation rate for individual proteins (16 non-structural proteins, 4 structural proteins and 6 accessory proteins) and Table 2 presents the results for individual functional domains as identified from the structural analysis and the literature. The complete results are available in S1 and S2 Tables. As seen from Table 1, many of the SARS-CoV-2 proteins show a statistically significant difference in mutation rate when compared to their corresponding backgrounds, with nine being under-mutated and ten being over-mutated. We see that the majority of under-mutated proteins are non-structural proteins (7 out of 9), which mirrors the trend seen earlier. This is seen despite the fact that individual non-structural proteins were compared to the set of all non-structural proteins as the background, and individual structural and accessory proteins were compared to the set of all structural and accessory proteins, suggesting that these proteins are under strong negative selection. Most of the non-structural proteins play a role in RNA replication/processing and are part of the viral replication and transcription complex (RTC) [23,24]. Their low mutation rate can be explained by the fact that these proteins are crucial for the viral life cycle. One of the exceptions to this trend is nsp1, with a mutation rate similar to that of orfs2-14. Indeed, its function is somewhat different from the other non-structural proteins, as it interacts with the ribosome to inhibit host protein translation [25,26]. Overall, 6 out of the 10 over-mutated proteins are structural and accessory proteins. Over-mutation generally implies lower negative selection or potentially some positive selection, and again, this can be explained for at least some of these proteins based on their functions, which involve interacting with components of the host cell. For example, Orf8, Orf6 and N protein have been implicated in disrupting the host anti-viral immune response [23,24] so their high mutation levels can contribute to the immune avoidance by the SARS-CoV-2 virus.

**Table 1:** SARS-CoV-2 proteins with a significantly different rate of mutations as compared to the corresponding background (set of non-structural proteins/set of structural and accessory proteins).

Protein name	Genomic start position	Genomic end position	Length (nt.)	No. of missense mutations	Expected no. of missense mutations	p-value	q-value (FDR corrected)
**Proteins under-mutated as compared to the background**							
nsp4	8555	10054	1500	770	885.71	4.20E-05	8.09E-05
nsp5	10055	10972	918	448	542.05	2.47E-05	5.12E-05
nsp8	12092	12685	594	307	350.74	1.71E-02	2.57E-02
nsp9	12686	13024	339	164	200.17	9.25E-03	1.47E-02
nsp10	13025	13441	417	196	246.23	9.12E-04	1.54E-03
nsp12	13442	16236	2796*	1283	1650.96	9.58E-24	6.46E-23
nsp13	16237	18039	1803	893	1064.62	1.86E-08	5.57E-08
membrane glycoprotein	26523	27191	669	318	523.7	1.44E-23	7.80E-23
surface glycoprotein	21563	25384	3822	2462	2991.87	1.54E-39	4.16E-38
**Proteins over-mutated as compared to the background**							
nsp1	266	805	540	465	318.86	8.02E-15	3.61E-14
nsp2	806	2719	1914	1525	1130.17	7.06E-32	9.53E-31
nsp3	2720	8554	5835	3746	3445.41	2.52E-09	8.50E-09
nsp15	19621	20658	1038	718	612.91	2.16E-05	4.87E-05
Orf3a protein	25393	26220	828	907	648.16	2.99E-24	2.69E-23
Orf6 protein	27202	27387	186	173	145.6	2.36E-02	3.36E-02
Orf7a protein	27394	27759	366	396	286.51	3.70E-10	1.43E-09
Orf8 protein	27894	28259	366	379	286.51	8.65E-08	2.12E-07
nucleocapsid phosphoprotein	28274	29533	1260	1147	986.33	5.41E-08	1.46E-07
Orf14 protein	28734	28955	222	226	173.78	1.18E-04	2.13E-04

*contains a single additional nucleotide because of ribosomal slippage, see Genbank entry for MN908947.3

**Table 2:** Structurally characterized protein domains with rate of mutations significantly different than the background (the encompassing full protein).

Protein	Domain name / pdb IDs	Genomic start	Genomic end	Domain length (nt.)	No. of missense mutations in domain	Protein length (nt.)	Expected no. of missense mutations in domain	p-value	q-value (FDR corrected)
**Domains under-mutated as compared to the background**									
nsp3	SUD (SARS Unique Domain) / 2w2gA	3956	4747	792	446	5835	508.45	2.64E-03	8.73E-03
nsp3	interdomain linker / Region b/w 6w9cA and 2k87A	5900	5983	84	35	5835	53.93	7.36E-03	1.52E-02
nsp3	Y1 domain / none	7469	8011	543	235	5835	348.60	1.96E-11	6.47E-10
nsp3	CoV-Y domain / none	8012	8554	543	299	5835	348.60	4.90E-03	1.24E-02
S	Receptor binding domain (RBD) / 6lzgB	22559	23143	585	299	3822	376.84	8.39E-06	4.61E-05
S	RBD assoc. linker domain / none	22529	22558	216	108	3822	139.14	5.91E-03	1.36E-02
		23144	23329						
S	6vxxB	21641	25003	3363	2060	3822	2166.33	2.81E-10	4.64E-09
N	RNA-binding domain / 6m3mA	28415	28792	378	273	1260	344.10	3.37E-06	2.22E-05
N	C-terminal dimerization domain / 6wjiA	29042	29365	324	246	1260	294.94	8.19E-04	3.00E-03
nsp4	C-terminal domain of nsp4 / 3vcbA	9782	10051	270	109	1500	138.60	4.86E-03	1.24E-02
**Domains over-mutated as compared to the background**									
nsp3	ubiquitin-like domain 1 (Ubl1) of nsp3 / 7kagA	2720	3040	321	248	5835	206.08	3.29E-03	9.86E-03
nsp3	ADP-ribose phosphatase domain (ADRP) / 6w02A	3341	3835	495	378	5835	317.78	5.96E-04	2.46E-03
nsp3	interdomain linker / Region b/w 6w02A and 2w2gA	3836	3955	120	134	5835	77.04	2.32E-09	1.92E-08
S	N-terminal domain (NTD) / none	21563	22435	873	690	3822	562.36	2.16E-09	1.92E-08
N	Region b/w 6m3mA and 6wjiA	28793	29041	249	285	1260	226.67	2.70E-05	1.27E-04
N	Region b/w 6wjiA and Nucleocapsid end	29366	29533	168	185	1260	152.93	6.20E-03	1.36E-02

In the next step, we looked at individual domains within SARS-CoV-2 proteins. As domains within multidomain proteins often have their independent evolutionary history and identifiable, individual functions, differences in mutation rates between different domains may provide a more detailed picture of their relative importance for the viability of the virus. We have used a similar approach in the eDriver algorithm used to identify the role of individual domains in cancer driver proteins [27] (with all the caveats discussed in the previous section). The expression of multiple constructs from individual proteins allowed researchers to recognize fragments that could fold independently and often can be assigned specific functions. Three-dimensional structures of many of these domains have been determined, so here we use the mapping of the SARS-CoV-2 proteins into the PDB structures/models as a proxy for identification of domain boundaries (Table 2), in addition to those identified through the literature.

**Fig 2:**
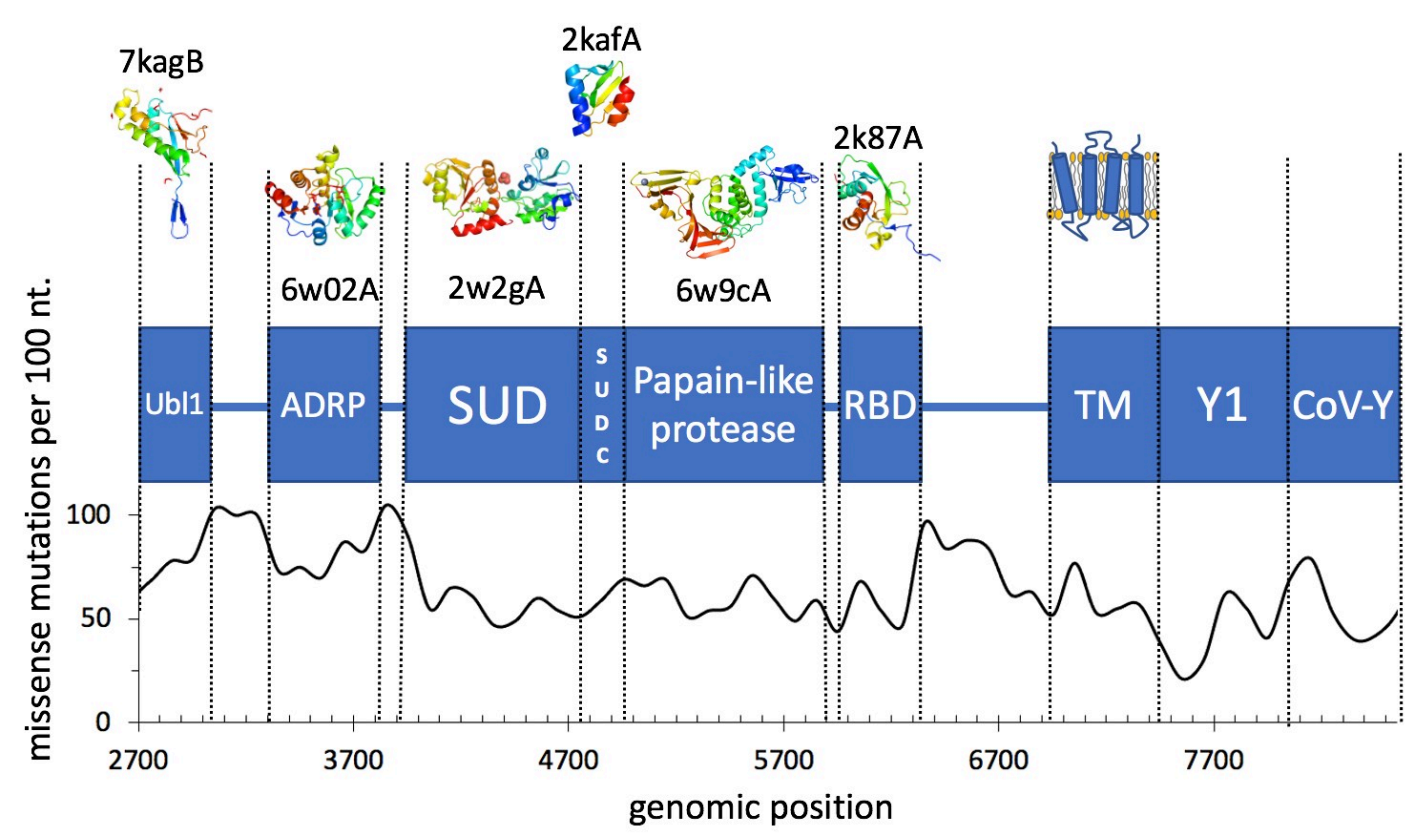
Domains and missense mutation rate per 100 nt window in the nsp3 protein.

We also looked at regions in between the solved structures/models, assuming that these would form important linker regions or domains whose structures remain unsolved (Fig 2 and Table 2). The complete list of domains found in SARS-CoV-2 proteins, and the relative excess or dearth of mutations in them, is provided in S2 Table.

We see domains with significantly different mutation frequencies in four proteins: nsp3, nsp4, N (nucleocapsid phosphoprotein) and S (spike/surface glycoprotein) protein. In N protein we see that the structured regions i.e., the RNA binding domain and the dimerization domain (PDB IDs 6m3mA and 6wjiA, respectively), are significantly under-mutated. This effect can probably be explained by the rest of the nucleocapsid protein (including the region between the two domains) being partly disordered, since such regions are typically much more tolerant of mutations (for the detailed discussion of mutation rate in different parts of the N protein–see the section *Significantly lower mutation rate in the region of overlapping reading frames*). In line with this, we see that the region between the domains is significantly over-mutated. In nsp3, three domains are over-mutated (Ubl1, ADRP and a linker domain) and four are under-mutated (SUD, another linker, Y1 and CoV-Y domains). The SUD domain, which is further composed of two macrodomains (Mac2 and Mac3), has been shown to bind to G-quadruplexes. This binding occurs via lysine residues in both macrodomains; however, it was shown through mutational analyses that only the lysine residues in Mac3 are essential for binding [28]. Moreover, this binding appears to be essential for viral replication [29], supporting the low mutation rate of this domain. The Ubl1 and ADRP domains have both been suggested to interfere with the host immune response. However, the connection between their functions and the observed mutation distribution is less clear, particularly since they may perform more than one function [22].

### Evidence of protein structure—driven purifying selection in SARS-CoV-2

**Fig 3:**
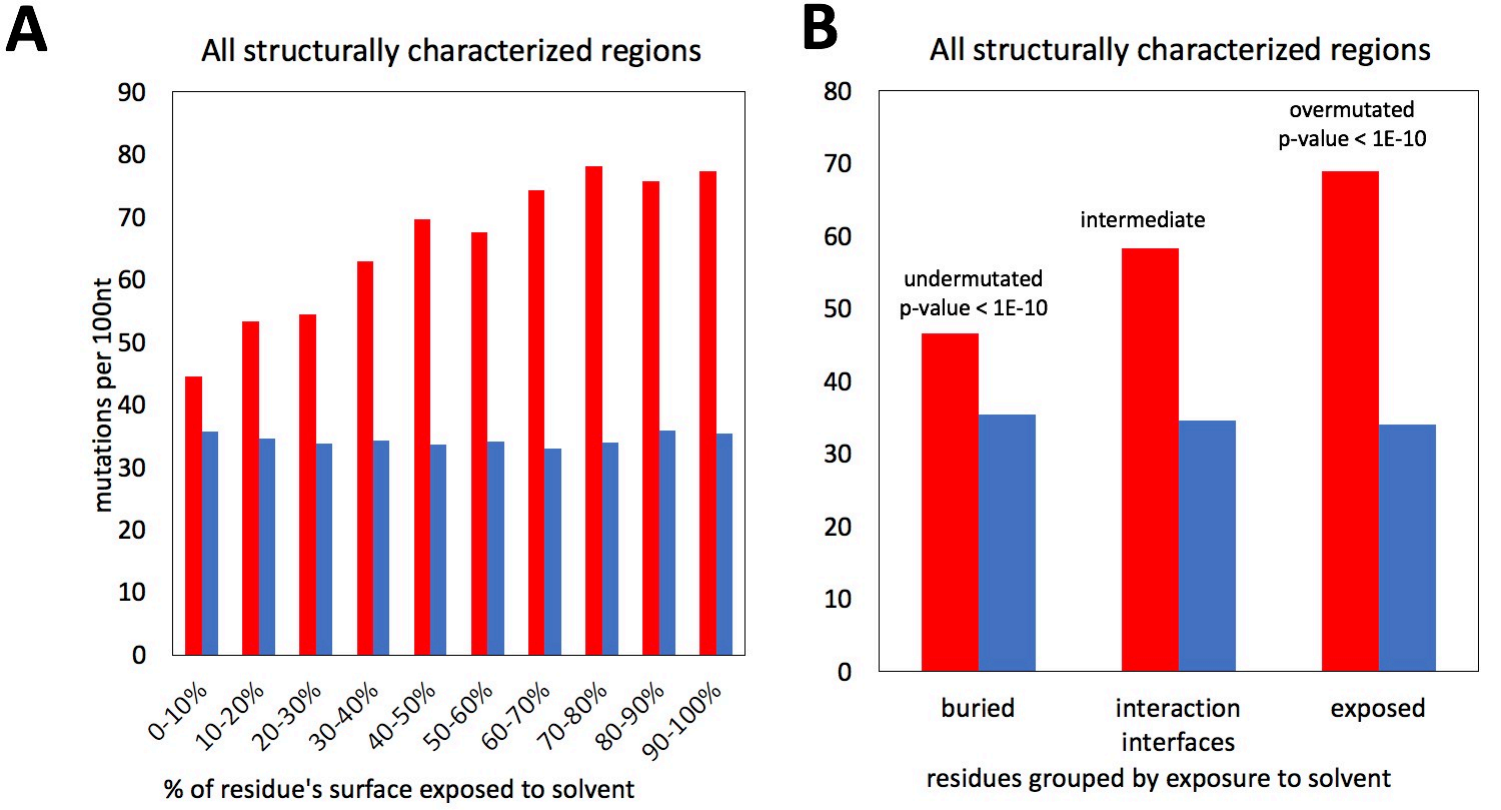
Frequencies of missense (red) and synonymous (blue) mutations for residues buried in the protein core, exposed to the solvent and involved in known protein-protein interfaces. **A)** The purifying selection decreases with increasing solvent exposure. **B)** Exposed residues are very over-mutated and buried residues are very under-mutated. For interfaces, the mutation rate falls between that for exposed and buried residues.

The proportion of missense mutations in structurally characterized protein residues of SARS-CoV-2 increases with their increasing solvent exposure following a known trend observed in many protein families from different organisms [10]. There is a strong, nearly linear increase in the rate of missense mutations, with synonymous mutations remaining at an approximately constant level, similar to their flat distribution along the genome discussed earlier (Fig 3A). The strong change in missense mutations rates is explained by tightly packed cores presenting strong constraints for amino acid residue choices and many mutations there leading to unfolded protein products. Protein-protein interaction interfaces do not pack as tightly as protein cores, but also have specific amino-acid composition and their mutations may lead to function-affecting changes in protein complex formation. Notably, in cancer, we see the opposite effect, with disproportionately high number of driver mutations found on protein-protein interfaces of cancer driver genes. SARS-CoV-2 non-structural proteins are known to form higher order assemblies essential for their function [30], thus, we can expect that in most cases interface residues should be conserved. This hypothesis is confirmed by the results shown in Fig 3B, where the ratio between missense and synonymous mutations for the residues on known protein interaction interfaces falls to a value between those for exposed and buried residues.

In the calculations shown here we only used the information on currently known protein-protein interfaces in SARS-CoV-2 proteins, based on experimental structures of viral protein complexes. We can expect that “unexplained” conserved patches on the surfaces of SARS-CoV-2 proteins may aid the discovery of some yet unknown interaction interfaces.

#### Significantly lower mutation rate in the region of overlapping reading frames

**Fig 4:**
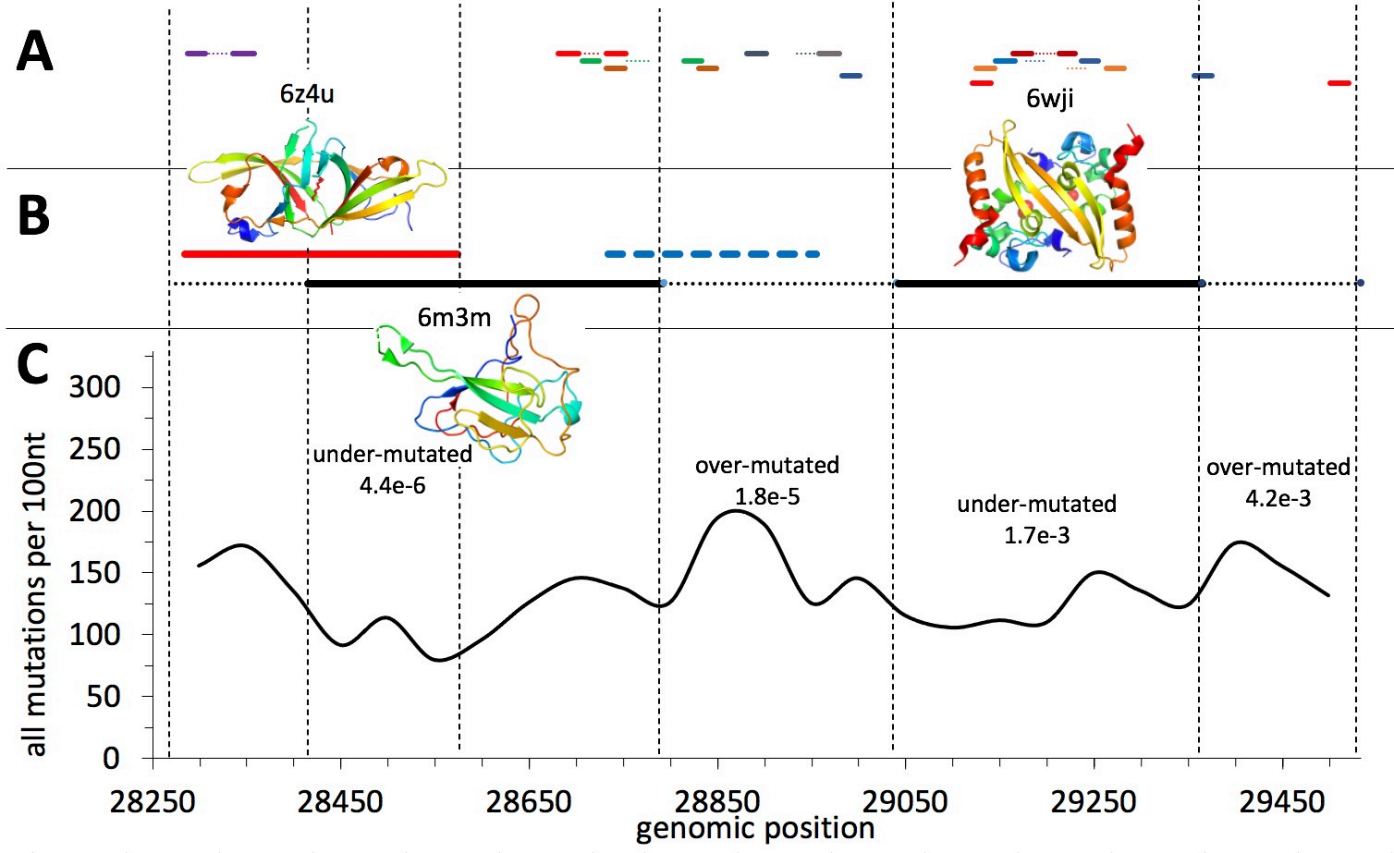
**A)** Regions of Nucleocapsid gene targeted by the diagnostic PCR-based tests. Primers are shown as continuous lines and probes–as dotted lines. Primers and the probe of the same test are shown in the same color. **B)** The open reading frames: Orf9b is shown in red, Orf14 –in blue and Nucleocapsid–in black. The regions coding for experimentally verified stable protein structures are shown as continuous lines and regions known to be structurally disordered—as dotted lines. Orf14, whose protein structure remains to be determined is shown as a dashed line. **C)** The rate of all (missense + synonymous) mutations per 100 nt windows. P-values from binomial tests are given for regions which are significantly under- or over-mutated (the entire Nucleocapsid ORF was used as a background).

Overlapping reading frames are common in viruses, resulting in local protein coding density over 100% [31]. Systematic analyses suggest that combined negative selection on two reading frames results in decreased rate of all mutations as mutations synonymous in one reading frame may be missense (and potentially deleterious) in another reading frame [32]. The N-terminal part of the Nucleocapsid gene of SARS-CoV-2 is translated into two different reading frames resulting in an additional gene coding for a functional protein Orf9b. A similar overlapping reading frame is suggested for the region coding for Orf14. We tested the rate of mutations in the region of the Nucleocapsid protein which is coding for two proteins in two different reading frames and compared the mutation rate in this region to the background rate for the entire gene confirming the expected result (see Fig 4). The largest decrease in the rate of mutations is observed in the region where proteins coded in two reading frames (Orf9b and Nucleocapsid) have well-defined structures. The N-terminal region of the Nucleocapsid gene does not have an experimental structure and is predicted to be structurally disordered and, as such, is expected to impose less constraints on mutations [33]. Despite the fact that it also overlaps with Orf9b (see Fig 4), the density of mutations there is not decreased.

The protein-coding Orf14 does show only moderate decrease in mutation density, in the region where it overlaps in a different reading frame with the gene coding for Nucleocapsid. This is probably again explained by the fact that it mostly overlaps with the structurally disordered region of the Nucleocapsid protein which does not impose strong constraints on missense mutations.

These observations have important practical implications for the selection of primers and probes for COVID-19 diagnostic tests as mutations in their target genomic regions have detrimental effect on their accuracy. Taking into account constraints on mutation rate imposed by protein structure and function may help in selecting regions which are less likely to accumulate mutations in the future. Unfortunately, in fact, multiple PCR-based diagnostic tests for COVID-19 target the genome region encoding the Nucleocapsid protein (see Fig 4) with some of them mapping to the highly mutated disordered protein regions. We discuss this issue in more detail in a separate section.

### Missense mutations in epitopes on the Receptor Binding Domain of the Spike protein

The Spike protein is the main surface antigen of SARS-CoV-2, a preferred target of therapeutic antibodies for COVID-19, and the immunogen used in the currently available vaccines. There are already more than 40 structurally characterized complexes of various types of antibodies with the Spike protein and almost all of them bind to its Receptor Binding Domain (RBD). Therefore, in the following analysis, we only focused on epitopes localized on the RBD.

Substitutions of residues in epitopes are a serious potential problem for both therapeutic antibodies and vaccines. At the same time, many of these epitopes overlap with the part of the RBD surface that binds to human ACE2 –the main entry receptor for SARS-CoV-2. The surfaces mediating interactions between SARS-CoV-2 proteins are under-mutated indicating purifying selection (Fig 3B) and therefore it can be expected that the RBD-ACE2 interface would also be under-mutated.

**Fig 5:**
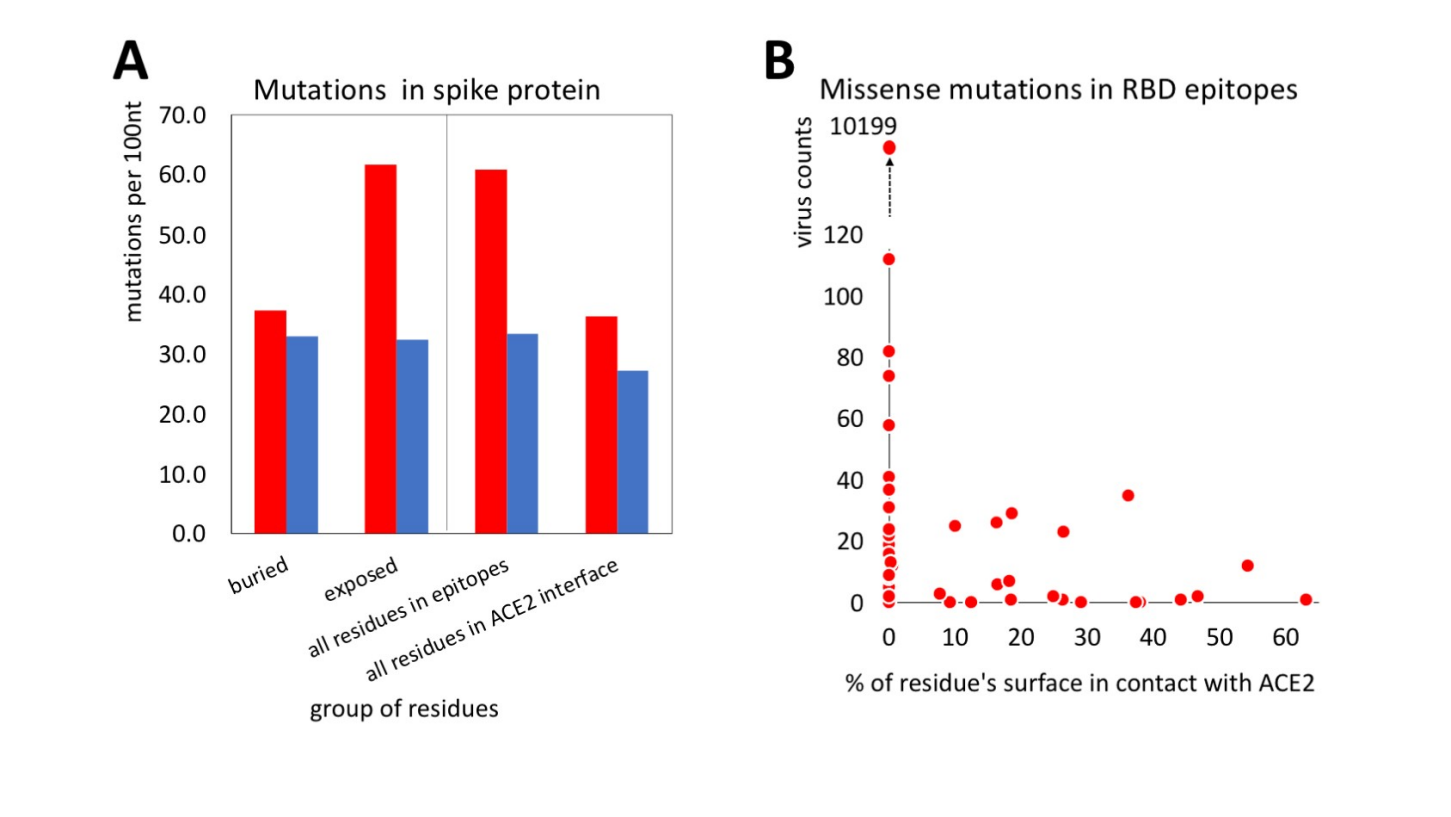
Mutations in known epitopes of the RBD of SARS-CoV-2 spike protein: **A)** Frequencies of missense (red) and synonymous (blue) mutations in (from the left to right): residues buried in the Spike protein core, in its exposed residues, in all residues from (structurally characterized) epitopes in RBD, and in all residues involved in binding to the human ACE2 receptor. **B)** The virus counts of missense mutations in epitopes as a function of the percent of residue’s surface that is involved in the RBD-ACE interface.

**Fig 6:**
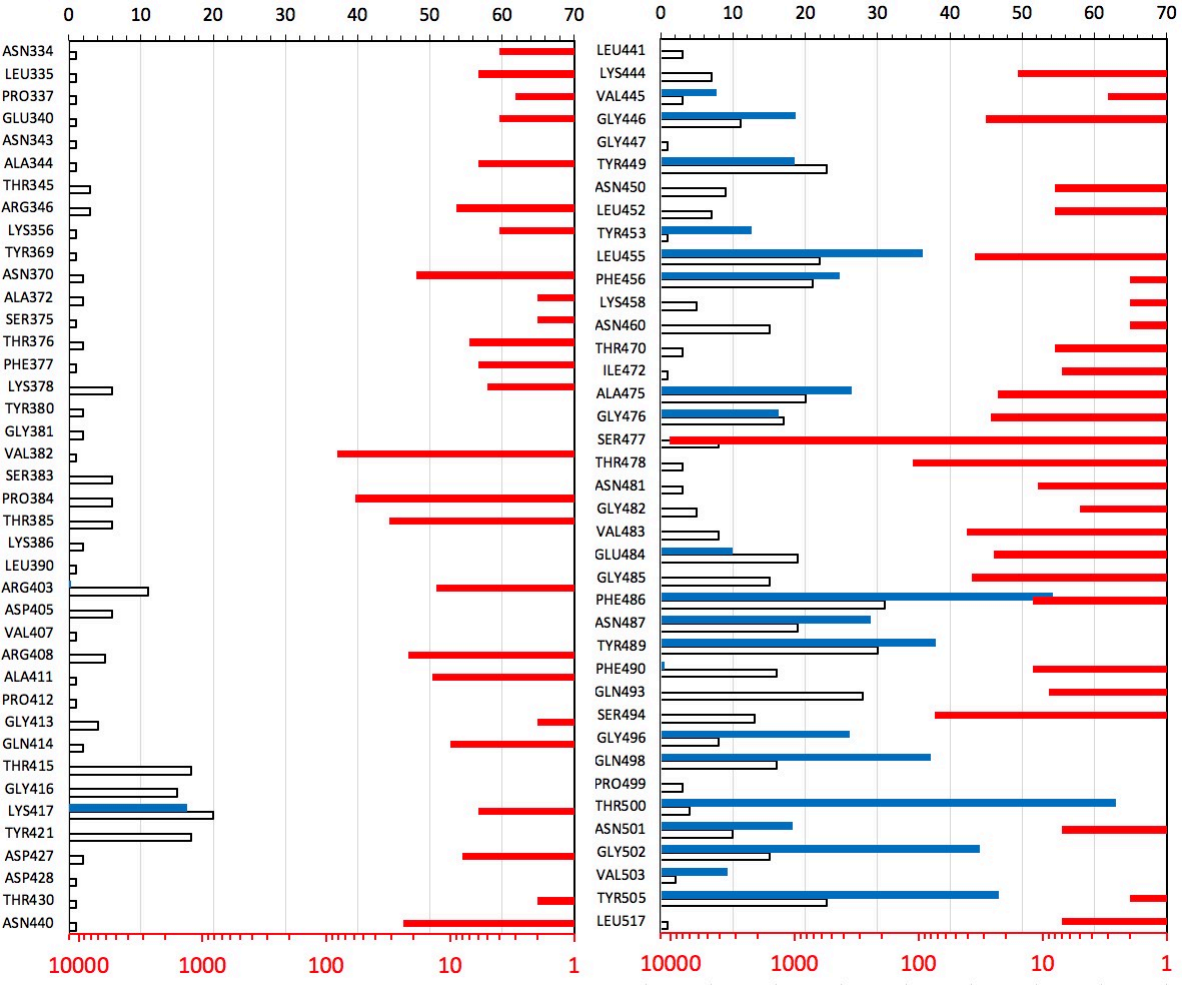
An overview of mutations in structurally characterized epitopes on RBD of SARS-CoV-2 spike protein. The percentages of residue’s area in contact with ACE2 are shown as blue bars. The number of epitopes a residue is involved in is shown as white bars. The prevalence of missense mutations in epitopes is shown as red bars on the opposite (logarithmic) scale. For more details on mutations in currently structurally characterized epitopes, see S2 Fig.

Indeed, the comparison of missense mutation rates in different groups of residues of the Spike protein trimer shows that the rate of missense mutations in epitopes is close to that of other exposed residues (see Fig 5A). However, exposed residues involved in the RBD-ACE2 interface appear to be under strong purifying selection as they are significantly under-mutated as compared to other exposed residues (p-value = 0.03). This is expected as the RBD-ACE2 interface is essential for the entry of the virus into the host cell and any, even minor, disruption of its binding would most likely diminish the ability of the virus to enter host cells. As a result, the epitope residues that are also involved in the RBD-ACE2 interface are effectively “protected” from mutations. It seems that mutations, especially those which are observed multiple times (higher virus counts), are unlikely to be found on this interface (Figs 5B and 6). In individual epitopes, the positions significantly involved in contact with ACE2 only rarely have mutations and these mutations usually have low viral counts (see Fig 6). While sufficient statistics for these trends are still lacking, they support the idea that antibodies targeting epitopes with large overlap with the ACE2 interaction interface are at a lower risk of immunological escape by the virus.

### The rate of mutations in regions targeted by the diagnostic PCR tests

The adverse effects of SARS-CoV-2 genomic mutations on the PCR-based diagnostic test results have already been discussed by others [34,35] (also see the GISAID page on popular primers available at https://www.gisaid.org/). The false-negative results of the PCR tests, especially of the TaqMan-qPCR assay are linked to mutations and the high sensitivity of this technique to primer/probe-template mismatches [36,37]. Both missense and synonymous mutations have an impact on the accuracy of PCR tests, but only missense mutations are under structural and functional constraints imposed by proteins. Nevertheless, since missense mutations comprise most (59%) of the mutations in the SARS-CoV-2 genome, the overall mutation rate depends mostly on missense mutations.

**Fig 7:**
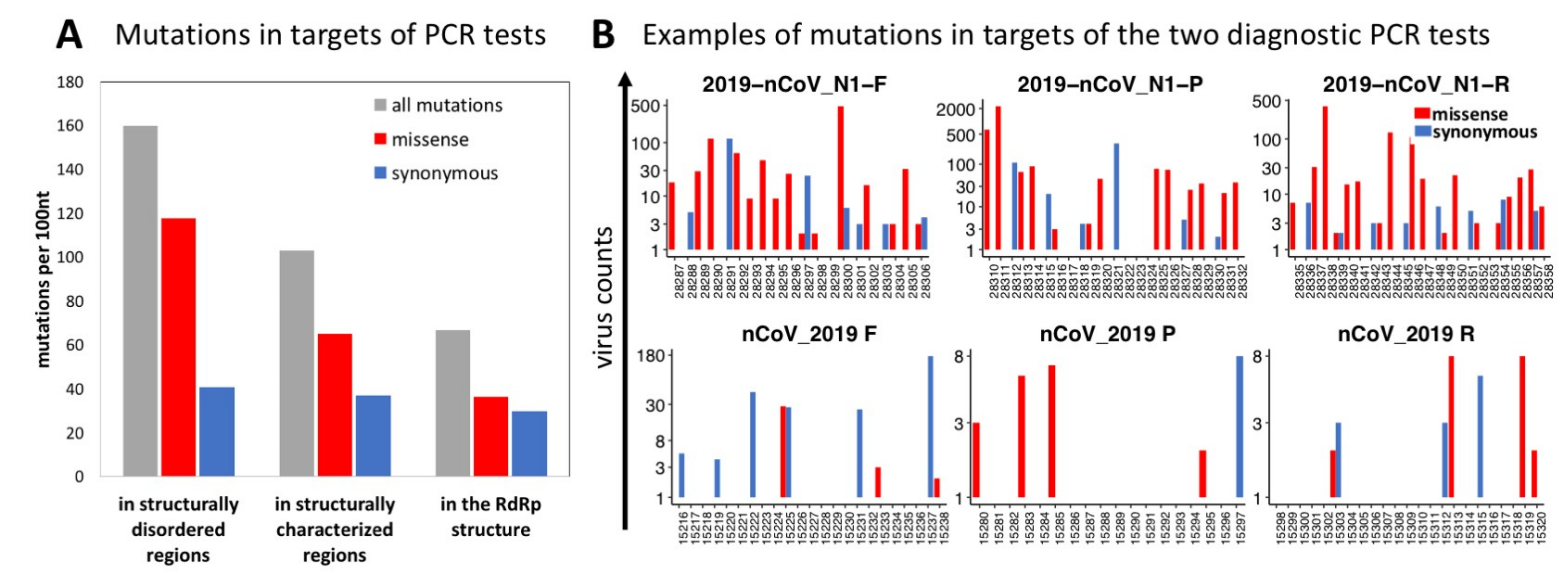
The frequencies of SARS-CoV-2 mutations in genomic regions targeted by the primers and probes of the diagnostic PCR tests **A)** The regions targeted by popular PCR tests have lower missense mutation rates when those regions are structurally characterized or map to the RdRP structure, and higher missense mutation rates when the regions are structurally disordered. On the other hand, the rate of synonymous mutations remains roughly the same. **B)** Examples of the effects of constraints imposed by encoded proteins on rates of mutations in regions targeted by the PCR tests. The region targeted by the 2019-nCoV_N1 PCR test (top) encodes the structurally disordered linker region of the Nucleocapsid protein. The region targeted by the nCoV_2019 PCR test (bottom) encodes the RNA-dependent RNA polymerase (RdRP). All reported counts are based on 192,030 high-coverage genomes obtained as of Dec 3^rd^, 2020 from the GISAID website.

Here we investigated the mutation rates of the target regions of the widely used PCR primers and probes in the context of proteins and protein domains encoded by these regions. To this end, we collected the sequences of primers and probes commonly used for COVID-19 diagnostic PCR assays. The coordinates of the genomic target regions of these primers and probes were obtained by mapping them to the reference genome used in this study (GenBank: MN908947.3) and then these genomic coordinates were mapped to SARS-CoV-2 proteins and (where possible) to experimental structures. As expected, the tests targeting the genomic regions encoding highly conserved proteins whose functions are essential to the viral life-cycle, such as RdRP, show the lowest rate of mutations (Fig 7A). More generally, target regions encoding stable, protein structures have lower mutation rates than those encoding structurally disordered protein regions. Regions coding for structurally disordered proteins are known to be enriched in mutations [33] and this applies to the regions targeted by some widely used diagnostic tests (Fig 7B). The examples of such frequently mutated target sequences are the targets of 2019-nCoV_N1 (also known as RX7038-N1 or CDC N1) primers and probe as shown in Fig 7B. These regions encode the structurally disordered region of the SARS-CoV-2 Nucleocapsid protein. Our predictions of structural disorder obtained using the Disopred program [38] were recently confirmed experimentally as it was shown that the SARS-CoV-2 Nucleocapsid protein is highly dynamic and contains three disordered regions [39]. Such regions are less suitable as targets of PCR-based diagnosis of SARS-CoV-2. At the same time, the region coding for RdRP has few mutations (Fig 7B) and, thus, is a more reliable target for SARS-CoV-2 diagnostic purposes. The list of diagnostic primers and probes, mutation counts in their target regions, and proteins encoded by these regions are provided in S4 Table.

## Discussion

In this manuscript, we have shown that the connection between the distribution of amino acid mutations and structures of the proteins encoded in the genome is clearly evident in the evolution patterns for the SARS-CoV-2 virus.

The rate of missense mutations significantly varies along the SARS-CoV-2 genome, while the rate of synonymous mutations shows much lower variability. This indicates that mutations are significantly impacted by the selection mechanisms on the protein level. A simple analysis of the rate of missense mutations along the genome reveals some strong maxima and minima. Some peaks of mutation rate are correlated with structurally disordered regions where structural constraints on amino-acid substitution are generally lower [33]. At the same time, some deep minima in mutation rate correspond to known essential regions of SARS-CoV-2 proteins whose functions put significant constraints on the possible mutations. At least one deep minimum in the rate of missense mutations corresponds to the structurally uncharacterized C-terminal domain of nsp3, suggesting that it has a well-defined structure whose conservation is essential for the viral life cycle. The analysis of mutation frequencies of individual proteins and domains further corroborated these observations.

Additionally, the mutations in SARS-CoV-2 proteins follow a known trend [9] with positions corresponding to the residues in protein cores mutated less often than those corresponding to solvent-exposed residues. Positions on the protein-protein interfaces present an intermediate case, but it is possible that the existence of some as yet unknown interaction interfaces complicates the analysis. This also opens up a possibility of searching for such interfaces by looking for patches of below-average sequence variability on protein surfaces. Another fascinating example of constraints imposed by protein structures on SARS-CoV-2 mutations are proteins encoded by overlapping reading frames. In agreement with trends observed earlier in bacteria [33], the region of the Nucleocapsid gene that codes for two different protein structures in two reading frames shows a significantly lowered rate of mutations.

The analysis of the mutation pattern in the SARS-CoV-2 virus is interesting from the evolutionary point of view but may also be of practical importance. For instance, it makes it possible to predict which of the currently known epitopes on the surfaces of SARS-CoV-2 proteins are more likely to undergo widespread mutations in the future. Similar predictions can be useful for regions targeted by primers and probes used in PCR-based diagnostic tests for COVID-19, as there is already evidence that the accuracy of some of these tests has been negatively affected by the accumulation of multiple mutations [40]). We show examples from both categories, where structure constrained mutation rates may differentiate between evolutionarily stable and unstable epitopes or probe sites, leading to antibodies less prone to viral escape and more reliable PCR-based diagnostic tests, respectively.

It is noteworthy that another study which addressed mostly positive selection in the regions of the SARS-CoV-2 genome [41] reports high conservation of the central RNA replication machinery (nsp6-nsp13), suggests both strong positive and negative selection for the Spike protein and high conservation of the Orf3a-N region. While our analysis addresses mostly negative selection, the last of these observations is not in agreement with ours as we report most of these genes in the Orf3a-N region to be over-mutated. We believe that the difference between these analyses is partly due to the different scope of the two studies (RNA level analysis [41] and protein level in our analyses) and partly due to the limited number of genomes used by Berrio *et al*. [41]. Currently, the rate of missense mutations in some parts of the Orf3a-N region approaches 3 mutations per nucleotide suggesting very low conservation for these proteins.

Our simple approach of analyzing the frequency of mutations in genomic regions coding for proteins or domains, originally applied to cancer [18,42], has its limitations. It was most appropriate when the number of known SARS-CoV-2 genomes (and the number of detected mutations) was relatively low. With rapidly accumulating data and increasing rates of recurrence, mutation counts (which, in the beginning, were most likely linked to founders’ effects) are expected to be increasingly correlated with fitness. However, at the time of finalizing this manuscript the rate of missense mutations was still a good measure of local negative selection as demonstrated by its expected strong dependence on the residues’ solvent exposure and anticipated high value in structurally disordered regions.

## Methods

### Rate of mutations definition

In our analysis we focus on the rates of missense mutations (with the exception of overlapping reading frames where we analyze all mutations). However, in figures we also often show numbers of synonymous mutations as the illustration of the fact that their distribution is mostly flat and (as expected) it does not significantly differ between regions defined by structural and functional features of proteins. In the manuscript we use the term “rate of mutations” for the number of distinct mutations in a given protein region or per 100 nt. This does not include virus counts (numbers of known genomes with a given mutation) so in our approach each mutation is counted only once. We provide a more detailed justification of this approach in the Results section.

### Data collection and curation

Sequences and metadata of complete SARS-CoV-2 genomes were retrieved from GISAID (https://www.gisaid.org/). as of December 3^rd^, 2020. All low-coverage genomes i.e., genomes containing less than 29,000 nt. and those containing more than 1% of undetermined nucleotides (Ns) based on GISAID cutoff, were removed. Several recent studies have shown that each coronavirus genome has median minority variants (either inter- or intra-host) ranged from 1–38 [43–45] and this median range recorded as roughly 1–10 for point mutations (substitutions) per sample [46]. The high rate of mutations could simply arise from sequencing errors as pointed out in the literature [46] (also see https://virological.org/t/issues-with-sars-cov-2-sequencing-data/473). Therefore, to avoid including spurious mutations, we excluded genomes with substitution exceeding a cutoff of 1.5 interquartile range (IQR) above the 3^rd^ quartile of substitution rates in all genomes.

This filtering procedure resulted in 192,030 genomes and this set was used in all calculations and analyses in this study. One of the early annotated and sequenced complete genomes of SARS-CoV-2 (GenBank: MN908947.3) was retrieved from The National Center for Biotechnology Information (NCBI) and used as a reference for all genomic coordinates and as a query in all alignments.

### Alignment, variant calling, and annotation

We calculated a multiple sequence alignment (MSA) of all high-coverage SARS-CoV-2 genomes using MAFFT version 7 (https://mafft.cbrc.jp/alignment/server/) with the default parameters. The MSA file was then processed using SNP-sites [47] and BCFtools version 1.9 [48] for variant calling and variant normalizations, respectively. In all analyses, we only considered single nucleotide substitutions involving unambiguous nucleotides (A, T, C, G). In the text we simply refer to them as “mutations”. All variations identified in this study along with the corresponding metadata are accessible via VarCoV application available at http://immunodb.org/varcov/.

To annotate variants, we used SnpEff (http://snpeff.sourceforge.net/). We used R package “vcfR” to manipulate and visualize variant calling format (VCF) data. The complete genome of SARS-CoV-2 (GenBank: MN908947.3) was used as a reference for genomic coordinates of proteins, protein structures, and models.

### Comparison of missense mutations rate in non-structural proteins (Orf1ab) and in the structural and accessory proteins

For each protein (except for the two very short peptides Orf3b and nsp11), we counted the total number of missense mutations (based on genomic positions) that were observed in at least one sample (that is, virus counts were ignored). If two mutations occurred at the same genomic position but resulted in different base substitutions, they were counted independently.

We then used the (two-sided) binomial test to compare:

1. the rate of missense mutations in Orf1ab (except nsp11) against the rate of missense mutations in the complete proteome (excluding Orf3b and nsp11).
2. The rate of missense mutations in the set of all structural and accessory proteins (except Orf3b) against the complete proteome.
3. The rate of synonymous mutations in Orf1ab to the full proteome.
4. The rate of synonymous mutations in the set of all structural and accessory proteins to the full proteome.

(The binomial tests were performed in a manner similar to the analysis of mutation rates in individual proteins, described below). We found that Orf1ab is significantly under-mutated, while the structural and accessory proteins are significantly over-mutated in both missense (p = 8.01x10^-75^) and synonymous mutations (p = 5.72x10^-6^). We further used Pearson’s Chi-squared test with Yates’ continuity correction (as implemented in R) to assess the significance of the difference between the proportion of missense and synonymous mutations in these two regions (p = 1.85x10^-12^). Because of these significant differences we decided to use separate backgrounds of Orf1ab and structural and accessory proteins to evaluate over- and under-mutation of individual proteins.

The full list of proteins used in this analysis is provided in the S1 Table.

### Assessing differences in rate of missense mutations in individual SARS-CoV-2 proteins and domains

For the assessment of differences in mutation frequencies of individual proteins and domains we compared the rate of missense mutations in these regions to the rate of missense mutations in some larger background region encompassing the protein/domain of interest.

We used the binomial test to identify individual proteins and domains that have significantly different mutation frequencies, when compared to an appropriate background mutation rate. This approach was used previously by our group in the eDriver algorithm [27] to evaluate the significance of differences in mutation rates between domains of cancer driver proteins.

The arguments for the binomial test, which are the number of successes, the number of trials, and the expected probability of success, were set as follows:

1. The number of successes was the observed number of missense mutations in the protein/domain being analyzed. This was counted as the total number of distinct missense mutations in that protein/domain observed in at least one sample. Therefore, virus counts (the number of samples where the mutation was observed) were ignored, since we assumed that, in most cases, these would not represent independent mutation events (see discussion of recurrence in the Results section). However, missense mutations that occurred at the same genomic position, but resulted in different base substitutions were counted independently.
2. The number of trials was the number of missense mutations in the background region used for comparison.
3. The expected probability of success (under the null hypothesis) was equal to the length of the protein/domain divided by the length of the background region.

All lengths were calculated in terms of genomic positions (i.e., the length of the genomic region coding for the protein/domain being analyzed). Missense mutations were also counted at the level of genomic positions.

The following approaches and background regions were used in the analyses of individual proteins and domains:

1. We used the set of all non-structural proteins (Orf1ab) (see note below) as the background for analysis of individual non-structural proteins, and the set of all structural and accessory proteins (see note below) as the background for the analysis of individual structural and accessory proteins.

The full list of proteins analyzed can be found in S1 Table. Note: Two very short peptides Orf3b and nsp11 coded in alternative reading frames (containing 9 bases and 38 bases respectively) were excluded from these analyses.

1. Domains were identified based on protein structures or models and through the literature. (For that purpose, only structures/models representing segments of the protein and not the full protein were considered.) We also considered regions in between known structures/models to represent domains as well. The full list of domains can be found in S2 Table. For each domain analyzed, the encompassing full protein was used as the reference background region.

### Structural coverage of the SARS-CoV-2 proteome and derived structural characteristics

The structural data for biological assemblies of SARS-CoV-2 was downloaded from Coronavirus3D server developed recently by our group[12]. The Coronavirus3D server provides links to experimental structures of SARS-CoV-2 proteins stored in PDB [13] and models of protein regions of SARS-CoV-2 for which direct structural characterization is still lacking. Models were calculated with Modeller [49] based on FFAS [50] alignments. For the purpose of this study, we prepared a non-redundant list of structures which included non-overlapping structures and models providing only one structural characterization for each residue where possible (with the exception of structures coded in two different reading frames). The list of structures and models used in this study is provided in S3 Table.

The collected experimental and modeled biological assemblies of SARS-CoV-2 proteins were used to calculate solvent exposure with the GetArea program [51]. Solvent exposure was calculated separately for biological assemblies and for isolated chains. The buried residues were defined as those with less than 20% of their surface exposed to the solvent according to GetArea. The remaining residues were classified as exposed. Interfaces were defined as a subset of residues whose solvent exposure decreased by at least 20% of their total area in biological assembly as compared to an isolated chain.

### Assessment of mutation frequencies as function of solvent exposure

The list of single nucleotide mutations in SARS-CoV-2 genomes (prepared as described in the section *Collection and curation of SARS-CoV-2 variation data*) was merged with the solvent exposure data prepared for residues of SARS-CoV-2 proteins (as described in the previous section). The total numbers of synonymous and non-synonymous mutations were then calculated for codons of protein residues for different ranges and categories of solvent exposure. The significance of differences in rate of missense mutations between buried, exposed, and interface residues was again assessed using binomial tests as described in previous sections with the entire proteome of SARS-CoV-2 used as the background.

### Rate of mutations in epitopes on RBD of Spike and in Spike-ACE2 interface

For these calculations we used the following definitions. Epitopes include residues whose solvent exposure decreases by at least 20% of their maximal solvent exposed area in the RBD-antibody complex as compared to RBD alone. Similarly, ACE2-contact area for any residue from RBD is the % of its solvent exposure lost when RBD is bound to ACE2. Antibody binding and ACE2 areas were derived from separate PDB entries. For the purpose of comparison all epitopes are shown in the same structural context of the RBD-ACE2 complex (PDB id 6m0j) rather than in the context of the antibody RBD complexes. However, RBD may undergo some conformational changes in complexes with antibodies.

### Rate of mutations in overlapping reading frames

In the overlapping reading frames, we tested differences in rate of all mutations rather than only missense mutations as mutations which are missense in one frame may be synonymous in other and vice versa. The significance of the changes in rate of all mutations in different regions of Orf9b and Nucleocapsid proteins was calculated using binomial tests in a way analogous to that used for individual proteins (see the previous section). For example, the number of all mutations in the region of the overlap of two structures coded in different reading frames (positions 28415–28574), the total number of mutations in Nucleocapsid and the ratio of the length of the overlap to the total length of Nucleocapsid were used as number of successes, number of trials and background probability in binomial tests, respectively. All lengths were calculated in terms of nucleotides.

## Supporting information

S1 Fig **A**) Distribution of SARS-CoV-2 mutations among genomic regions. **B**) Distribution of SARS-CoV-2 mutations according to the type of annotation. SARS-CoV-2 mutations were called using the multiple sequence alignment of 192,030 high quality genomes (GISAID as of December 3^rd^, 2020) and annotated using SnpEff.
(TIFF)

S2 Fig The virus counts for missense mutations in residues of structurally characterized epitopes on the RBD of Spike protein of SARS-CoV-2 (red bars) and percentages of each residue’s surface involved in RBD-ACE2 interaction interface (green bars).
(PDF)

S1 Table The list of proteins analyzed using binomial tests for significant over- or under-mutation. (DOCX)

S2 Table The list of domains analyzed using binomial tests for significant over- or under-mutation. (DOCX)

S3 Table The list of structures and models of biological assemblies used for structural coverage of SARS-CoV-2 proteome. (DOCX)

S4 Table The list of widely used PCR tests for diagnosis of SARS-CoV-2 along with mutation counts in their target regions and proteins coded by these regions. (DOCX)
