# Supplementary figures and images for: The interplay of SARS-CoV-2 evolution and constraints imposed by the structure and functionality of its proteins
Source: PLoS Comput Biol. 2021 Jul 8;17(7):e1009147. doi: 10.1371/journal.pcbi.1009147 (PMC8291704; doi:10.1371/journal.pcbi.1009147)

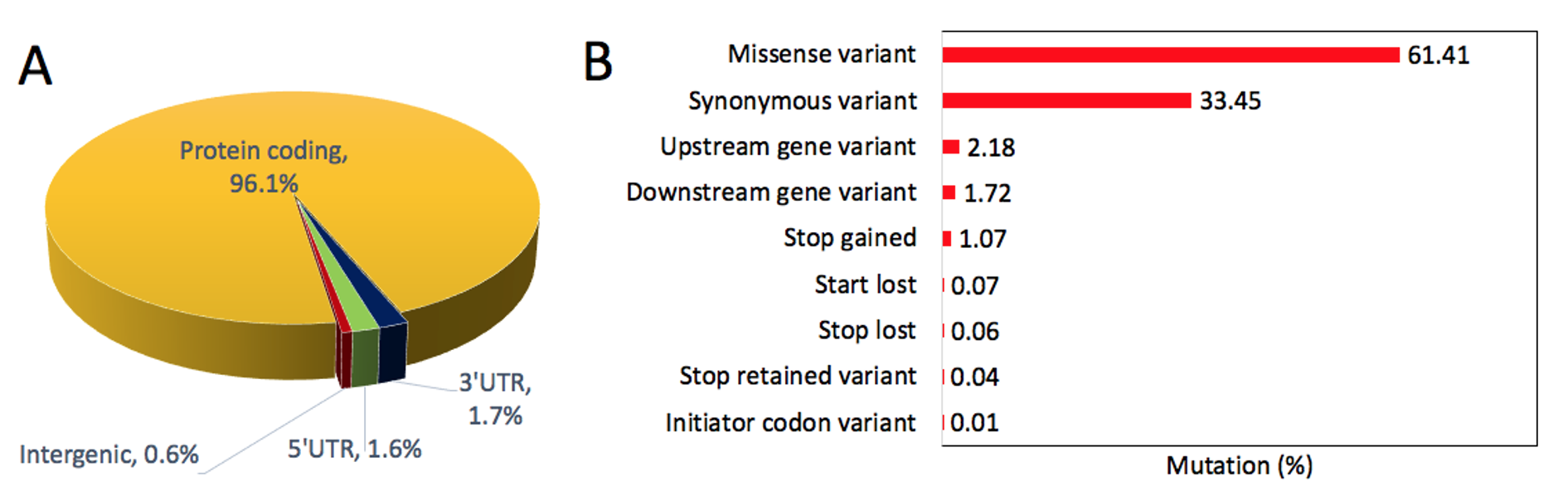

Supplement: S1 Fig — A) Distribution of SARS-CoV-2 mutations among genomic regions. B) Distribution of SARS-CoV-2 mutations according to the type of annotation. SARS-CoV-2 mutations were called using the multiple sequence alignment of 192,030 high quality genomes (GISAID as of December 3rd, 2020) and annotated using SnpEff. (TIFF) [file pcbi.1009147.s001.tiff]

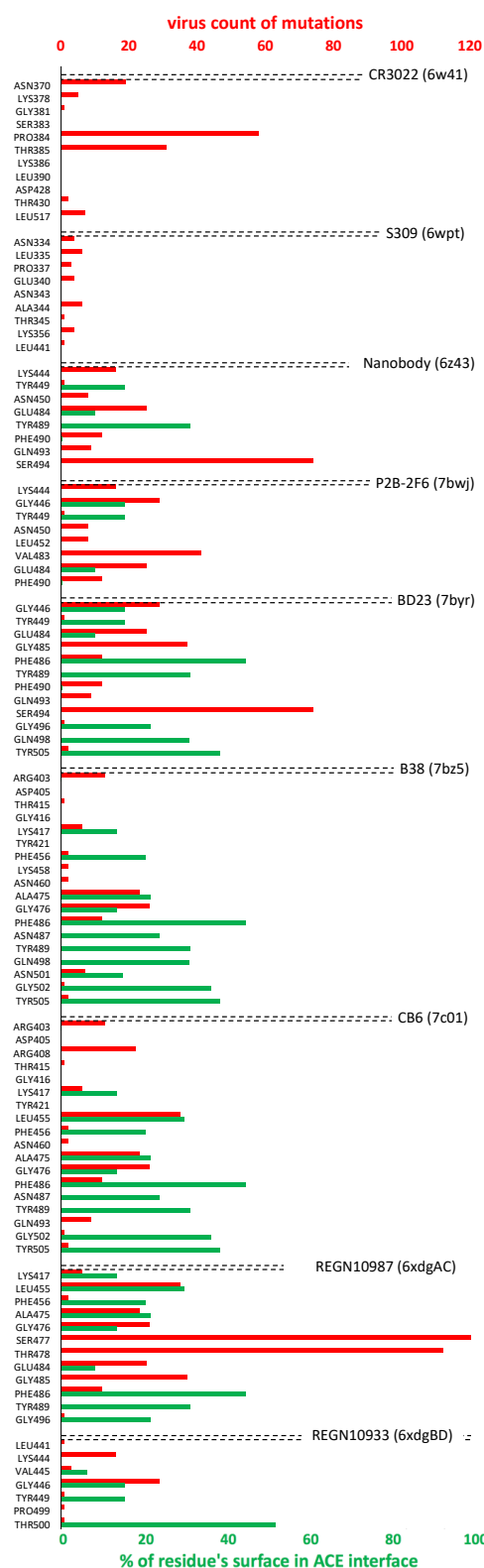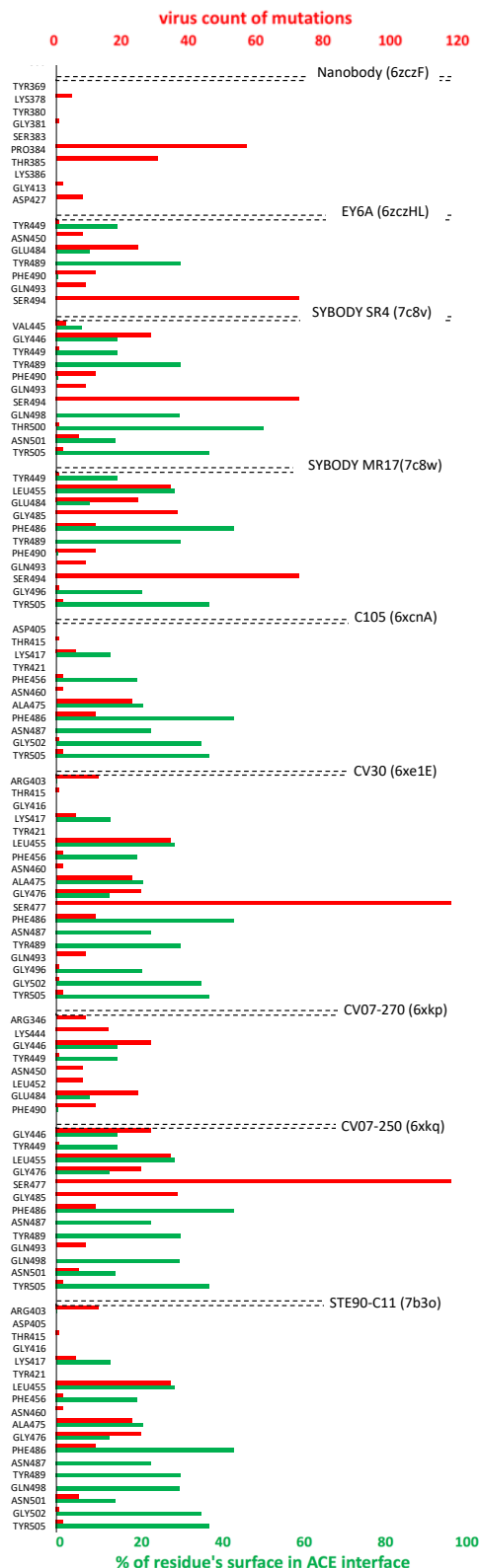

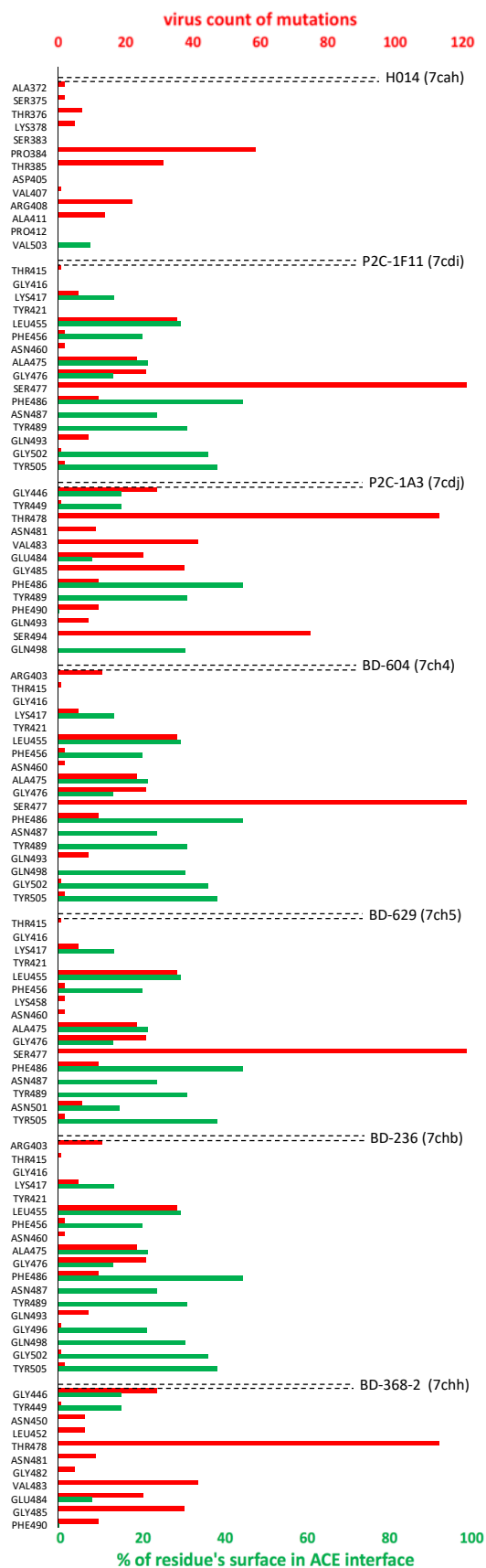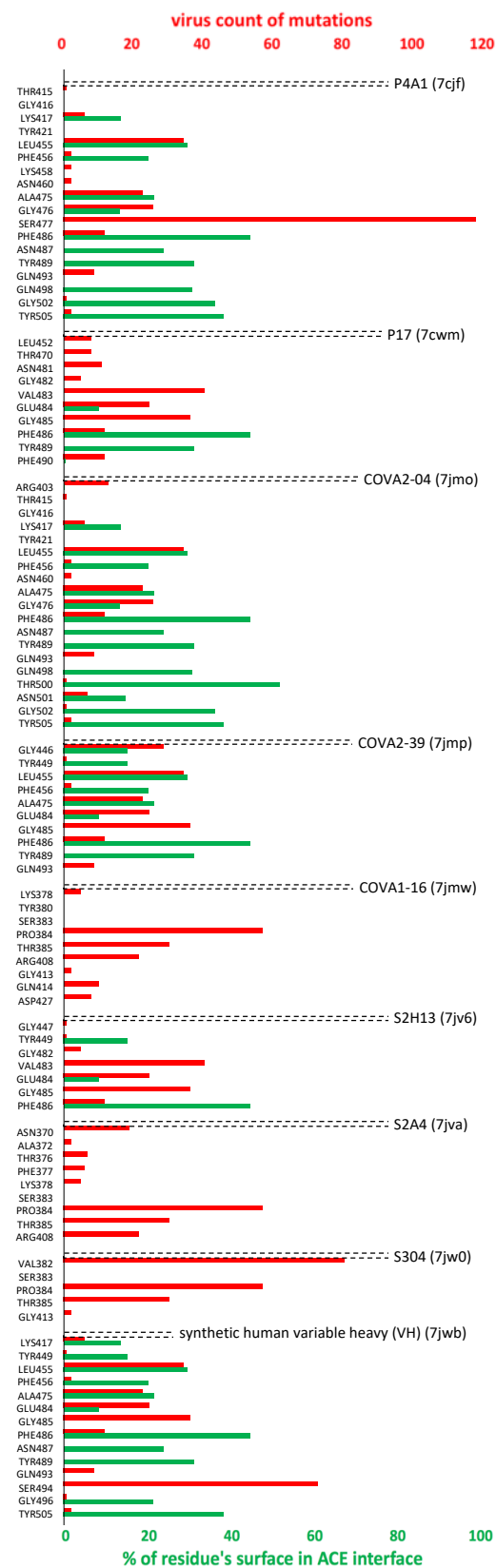

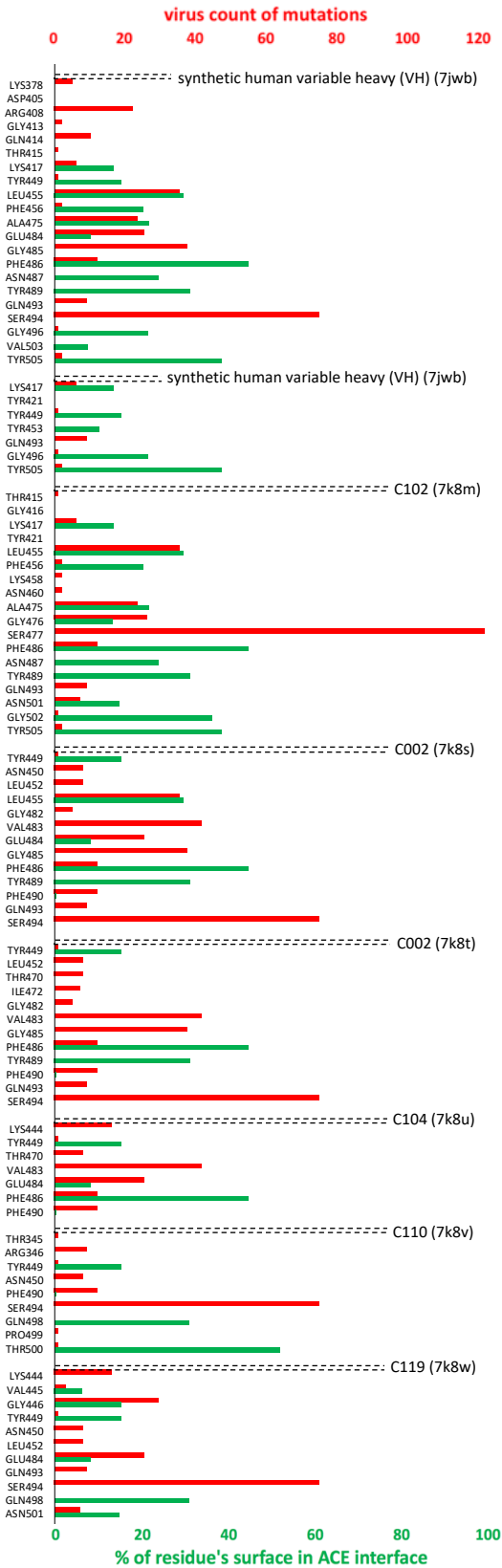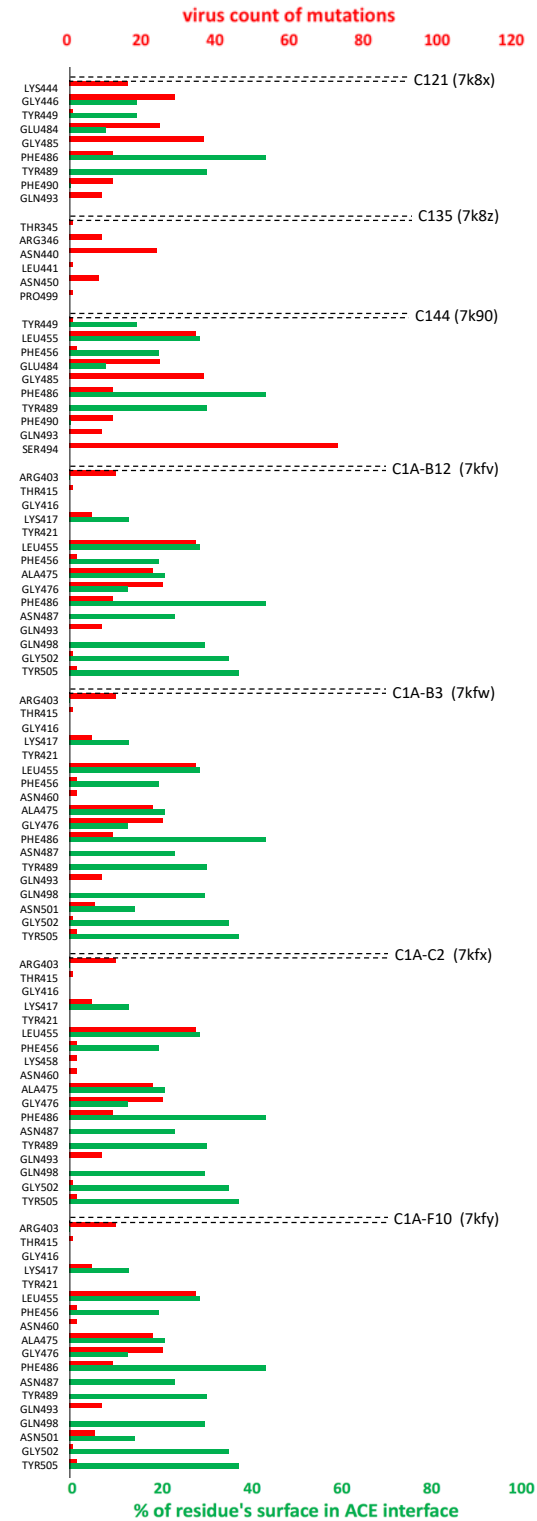

Supplement: S2 Fig — The virus counts for missense mutations in residues of structurally characterized epitopes on the RBD of Spike protein of SARS-CoV-2 (red bars) and percentages of each residue’s surface involved in RBD-ACE2 interaction interface (green bars). (PDF) [file pcbi.1009147.s002.pdf]
