# Supplementary material for: The interplay of SARS-CoV-2 evolution and constraints imposed by the structure and functionality of its proteins
Source: PLoS Comput Biol. 2021 Jul 8;17(7):e1009147. doi: 10.1371/journal.pcbi.1009147 (PMC8291704; doi:10.1371/journal.pcbi.1009147)
Supplement: S1 Table — (DOCX) [file pcbi.1009147.s003.docx]

| **Protein name** | **Genomic start** | **Genomic end** | **Length (nt.)** | **No. of missense mutations** | **Expected no. of missense mutations** | **p-value** | **q-value (FDR correction)** |
| --- | --- | --- | --- | --- | --- | --- | --- |
| nsp1 | 266 | 805 | 540 | 465 | 318.86 | 8.02E-15 | 3.61E-14 |
| nsp2 | 806 | 2719 | 1914 | 1525 | 1130.17 | 7.06E-32 | 9.53E-31 |
| nsp3 | 2720 | 8554 | 5835 | 3746 | 3445.41 | 2.52E-09 | 8.50E-09 |
| nsp4 | 8555 | 10054 | 1500 | 770 | 885.71 | 4.20E-05 | 8.09E-05 |
| nsp5 | 10055 | 10972 | 918 | 448 | 542.05 | 2.47E-05 | 5.12E-05 |
| nsp6 | 10973 | 11842 | 870 | 479 | 513.71 | 1.20E-01 | 1.41E-01 |
| nsp7 | 11843 | 12091 | 249 | 144 | 147.03 | 8.36E-01 | 8.36E-01 |
| nsp8 | 12092 | 12685 | 594 | 307 | 350.74 | 1.71E-02 | 2.57E-02 |
| nsp9 | 12686 | 13024 | 339 | 164 | 200.17 | 9.25E-03 | 1.47E-02 |
| nsp10 | 13025 | 13441 | 417 | 196 | 246.23 | 9.12E-04 | 1.54E-03 |
| nsp12 | 13442 | 16236 | 2796* | 1283 | 1650.96 | 9.58E-24 | 6.46E-23 |
| nsp13 | 16237 | 18039 | 1803 | 893 | 1064.62 | 1.86E-08 | 5.57E-08 |
| nsp14 | 18040 | 19620 | 1581 | 944 | 933.54 | 7.21E-01 | 7.49E-01 |
| nsp15 | 19621 | 20658 | 1038 | 718 | 612.91 | 2.16E-05 | 4.87E-05 |
| nsp16 | 20659 | 21552 | 894 | 488 | 527.88 | 7.89E-02 | 1.06E-01 |
| Orf3a protein | 25393 | 26220 | 828 | 907 | 648.16 | 2.99E-24 | 2.69E-23 |
| envelope protein | 26245 | 26472 | 228 | 162 | 178.48 | 2.25E-01 | 2.53E-01 |
| membrane glycoprotein | 26523 | 27191 | 669 | 318 | 523.7 | 1.44E-23 | 7.80E-23 |
| Orf6 protein | 27202 | 27387 | 186 | 173 | 145.6 | 2.36E-02 | 3.36E-02 |
| Orf7a protein | 27394 | 27759 | 366 | 396 | 286.51 | 3.70E-10 | 1.43E-09 |
| Orf7b protein | 27756 | 27887 | 132 | 121 | 103.33 | 8.25E-02 | 1.06E-01 |
| Orf8 protein | 27894 | 28259 | 366 | 379 | 286.51 | 8.65E-08 | 2.12E-07 |
| nucleocapsid phosphoprotein | 28274 | 29533 | 1260 | 1147 | 986.33 | 5.41E-08 | 1.46E-07 |
| Orf9b protein | 28284 | 28577 | 294 | 256 | 230.14 | 8.70E-02 | 1.07E-01 |
| Orf14 protein | 28734 | 28955 | 222 | 226 | 173.78 | 1.18E-04 | 2.13E-04 |
| Orf10 protein | 29558 | 29674 | 117 | 99 | 91.59 | 4.30E-01 | 4.64E-01 |
| surface glycoprotein | 21563 | 25384 | 3822 | 2462 | 2991.87 | 1.54E-39 | 4.16E-38 |
