# Supplementary material for: The interplay of SARS-CoV-2 evolution and constraints imposed by the structure and functionality of its proteins
Source: PLoS Comput Biol. 2021 Jul 8;17(7):e1009147. doi: 10.1371/journal.pcbi.1009147 (PMC8291704; doi:10.1371/journal.pcbi.1009147)
Supplement: S2 Table — (DOCX) [file pcbi.1009147.s004.docx]

| **Protein** | **PDB ID(s)** | **Domain** | **Genomic start** | **Genomic end** | **Domain length (in nucleotides)** | **No. of missense mutations in domain** | **No. of missense mutations in protein** | **Protein length (in nucleotides)** | **Expected no. of missense mutations in domain** | **p-value** | **q-value (based on FDR correction)** |
| --- | --- | --- | --- | --- | --- | --- | --- | --- | --- | --- | --- |
| nsp1 | 7k3nA | nsp1 head, N-terminal domain | 305 | 640 | 336 | 293 | 465 | 540 | 289.33 | 7.38E-01 | 8.12E-01 |
| nsp1 | none | nsp1 linker | 641 | 709 | 69 | 70 | 465 | 540 | 59.42 | 1.44E-01 | 2.38E-01 |
| nsp1 | 7k5i1 | plug domain, C-terminal domain | 710 | 799 | 90 | 68 | 465 | 540 | 77.5 | 2.63E-01 | 4.13E-01 |
| nsp3 | 7kagA | ubiquitin-like domain 1 (Ubl1) of Nsp3 | 2720 | 3040 | 321 | 248 | 3746 | 5835 | 206.08 | 3.29E-03 | 9.86E-03 |
| nsp3 | 6w02A | ADP-ribose phosphatase domain (ADRP) | 3341 | 3835 | 495 | 378 | 3746 | 5835 | 317.78 | 5.96E-04 | 2.46E-03 |
| nsp3 | Region b/w 6w02A and 2w2gA | interdomain linker | 3836 | 3955 | 120 | 134 | 3746 | 5835 | 77.04 | 2.32E-09 | 1.92E-08 |
| nsp3 | 2w2gA | SUD (SARS Unique Domain) | 3956 | 4747 | 792 | 446 | 3746 | 5835 | 508.45 | 2.64E-03 | 8.73E-03 |
| nsp3 | 2kafA | SARS-unique domain-C | 4754 | 4948 | 195 | 125 | 3746 | 5835 | 125.19 | 1.00E+00 | 1.00E+00 |
| nsp3 | 6w9cA | papain-like protease | 4961 | 5899 | 939 | 558 | 3746 | 5835 | 602.83 | 4.78E-02 | 8.77E-02 |
| nsp3 | Region b/w 6w9cA and 2k87A | interdomain linker | 5900 | 5983 | 84 | 35 | 3746 | 5835 | 53.93 | 7.36E-03 | 1.52E-02 |
| nsp3 | 2k87A | RNA binding domain | 5984 | 6328 | 345 | 207 | 3746 | 5835 | 221.49 | 3.32E-01 | 4.84E-01 |
| nsp3 | none | TM domain | 6956 | 7468 | 513 | 291 | 3746 | 5835 | 329.34 | 2.63E-02 | 5.11E-02 |
| nsp3 | none | Y1 domain | 7469 | 8011 | 543 | 235 | 3746 | 5835 | 348.6 | 1.96E-11 | 6.47E-10 |
| nsp3 | none | CoV-Y domain | 8012 | 8554 | 543 | 299 | 3746 | 5835 | 348.6 | 4.90E-03 | 1.24E-02 |
| nsp4 | 3vcbA | C-terminal domain of nsp4 | 9782 | 10051 | 270 | 109 | 770 | 1500 | 138.6 | 4.86E-03 | 1.24E-02 |
| nsp7 | 6xezC | nsp7 | 11843 | 12061 | 219 | 124 | 144 | 249 | 126.65 | 5.21E-01 | 6.61E-01 |
| nsp8 | 6xezB | nsp8 | 12107 | 12664 | 558 | 293 | 307 | 594 | 288.39 | 3.37E-01 | 4.84E-01 |
| nsp10 | 6w4hB | nsp10 | 13076 | 13423 | 348 | 162 | 196 | 417 | 163.57 | 7.73E-01 | 8.23E-01 |
| nsp12 | 6xezA | nsp12 | 13451 | 16227 | 2777 | 1274 | 1283 | 2796 | 1274.28 | 8.64E-01 | 8.91E-01 |
| S | none | N-terminal domain (NTD) | 21563 | 22435 | 873 | 690 | 2462 | 3822 | 562.36 | 2.16E-09 | 1.92E-08 |
| S | none | NTD assoc. linker domain | 22436 | 22528 | 420 | 256 | 2462 | 3822 | 270.55 | 3.67E-01 | 4.94E-01 |
|  |  |  | 23330 | 23656 |  |  |  |  |  |  |  |
| S | 6lzgB | Receptor binding domain (RBD) | 22559 | 23143 | 585 | 299 | 2462 | 3822 | 376.84 | 8.39E-06 | 4.61E-05 |
| S | none | RBD assoc. linker domain | 22529 | 22558 | 216 | 108 | 2462 | 3822 | 139.14 | 5.91E-03 | 1.36E-02 |
|  |  |  | 23144 | 23329 |  |  |  |  |  |  |  |
| S | none | S2 subunit | 23690 | 25384 | 1695 | 1081 | 2462 | 3822 | 1091.86 | 6.70E-01 | 7.90E-01 |
| 3a | 6xdcA | - | 25510 | 26106 | 597 | 642 | 907 | 828 | 653.96 | 3.74E-01 | 4.94E-01 |
| E | 5x29A | - | 26266 | 26439 | 174 | 113 | 162 | 228 | 123.63 | 5.25E-02 | 9.11E-02 |
| 7a | 6w37A | - | 27439 | 27636 | 198 | 209 | 396 | 366 | 214.23 | 6.14E-01 | 7.51E-01 |
| 8 | 7jx6A | - | 27945 | 28256 | 312 | 326 | 379 | 366 | 323.08 | 7.18E-01 | 8.12E-01 |
| N | 6m3mA | RNA-binding domain | 28415 | 28792 | 378 | 273 | 1147 | 1260 | 344.1 | 3.37E-06 | 2.22E-05 |
| N | Region b/w 6m3mA and 6wjiA | - | 28793 | 29041 | 249 | 285 | 1147 | 1260 | 226.67 | 2.70E-05 | 1.27E-04 |
| N | 6wjiA | C-terminal dimerization domain | 29042 | 29365 | 324 | 246 | 1147 | 1260 | 294.94 | 8.19E-04 | 3.00E-03 |
| N | Region b/w 6wjiA and Nucleocapsid end | - | 29366 | 29533 | 168 | 185 | 1147 | 1260 | 152.93 | 6.20E-03 | 1.36E-02 |
| S | 6vxxB | - | 21641 | 25003 | 3363 | 2060 | 2462 | 3822 | 2166.33 | 2.81E-10 | 4.64E-09 |
