# Supplementary material for: The interplay of SARS-CoV-2 evolution and constraints imposed by the structure and functionality of its proteins
Source: PLoS Comput Biol. 2021 Jul 8;17(7):e1009147. doi: 10.1371/journal.pcbi.1009147 (PMC8291704; doi:10.1371/journal.pcbi.1009147)
Supplement: S3 Table — (DOCX) [file pcbi.1009147.s005.docx]

| **PDB Id of a structure or a modelling template** | **Genomic start** | **Genomic end** | **Protein name** | **Protein function** | **Is it a model?** |
| --- | --- | --- | --- | --- | --- |
| 7k3nA | 305 | 640 | nsp1 | Host translation inhibitor nsp1 |  |
| 7k5i1 | 710 | 799 | nsp1 | nsp1 in complex with human 40S ribosome |  |
| 7kagA | 2720 | 3040 | nsp3 | RNA-binding domain |  |
| 6w02A | 3341 | 3835 | nsp3 | ADP ribose phosphatase |  |
| 2w2gA | 3956 | 4747 | nsp3 | RNA-binding domain | yes, template from SARS |
| 2kafA | 4754 | 4948 | nsp3 | SARS-unique domain-C from the non-structural protein 3 | yes, template from SARS |
| 6w9cA | 4961 | 5899 | nsp3 | Papain-like protease |  |
| 2k87A | 5984 | 6328 | nsp3 | RNA-binding domain | yes, template from SARS |
| 3vcbA | 9782 | 10051 | nsp4 | Cytoplasmic domain of TM protein | yes, template from MHV A59 |
| 5r82A | 10055 | 10966 | nsp5 | Protease |  |
| 6xezC | 11843 | 12061 | nsp7 | RNA-dependent RNA polymerase cofactor |  |
| 6xezB | 12107 | 12664 | nsp8 | RNA-dependent RNA polymerase cofactor |  |
| 6w4bA | 12698 | 13024 | nsp9 | Replicase |  |
| 6w4hB | 13076 | 13423 | nsp10 | Growth factor-like peptide |  |
| 6xezA | 13468 | 16277 | nsp12 | RNA-directed RNA polymerase |  |
| 6xezE | 16237 | 18024 | nsp13 | Helicase |  |
| 5nfyA | 18046 | 19614 | nsp14 | Proofreading exoribonuclease | yes, template from SARS |
| 6w01A | 19621 | 20655 | nsp15 | Uridylate-specific endoribonuclease |  |
| 6w4hA | 20659 | 21552 | nsp16 | 2'-O-methyltransferase |  |
| 6vxxB | 21641 | 25003 | S | Spike |  |
| 6lvnA | 25067 | 25168 | S | Spike HR2 domain |  |
| 6xdcA | 25510 | 26106 | 3a | Viroporin; modulation of virus release |  |
| 5x29A | 26266 | 26439 | E | Envelope, pentameric ion channel | yes, template from SARS |
| 6w37A | 27439 | 27636 | 7a | Accessory protein |  |
| 7jx6A | 27945 | 28256 | 8 | Orf8 |  |
| 6z4uA | 28284 | 28574 | 9b | Membrane attachment |  |
| 6m3mA | 28415 | 28792 | N | RNA binding domain of nucleocapsid phosphoprotein |  |
| 6wjiA | 29042 | 29365 | N | Dimerization domain of nucleocapsid phosphoprotein |  |
