# Supplementary material for: The interplay of SARS-CoV-2 evolution and constraints imposed by the structure and functionality of its proteins
Source: PLoS Comput Biol. 2021 Jul 8;17(7):e1009147. doi: 10.1371/journal.pcbi.1009147 (PMC8291704; doi:10.1371/journal.pcbi.1009147)
Supplement: S4 Table — (DOCX) [file pcbi.1009147.s006.docx]

| **Primer/probe Name** | **Total Pos with at least one missense mutation** | **Total Pos with at least one synonymous mutation** | **Maximum virus count** | **Genome Start** | **Genome stop** | **Protein(s)** |
| --- | --- | --- | --- | --- | --- | --- |
| 2019-nCoV_N1-F | 28 | 9 | 434 | 28287 | 28306 | N,9b |
| 2019-nCoV_N1-P | 23 | 7 | 2244 | 28310 | 28332 | N,9b |
| 2019-nCoV_N1-R | 31 | 10 | 390 | 28358 | 28335 | N,9b |
| 2019-nCoV_N2-F | 15 | 7 | 1154 | 29164 | 29183 | N |
| 2019-nCoV_N2-P | 16 | 11 | 353 | 29188 | 29210 | N |
| 2019-nCoV_N2-R | 11 | 6 | 2890 | 29230 | 29213 | N |
| CDC N3 Forward | 21 | 12 | 538 | 28681 | 28702 | N |
| CDC N3 Probe | 23 | 9 | 633 | 28704 | 28727 | N |
| CDC N3 Reverse | 16 | 12 | 116 | 28752 | 28732 | N |
| Ch_N_F | 36 | 10 | 67231 | 28881 | 28902 | N,14 |
| Ch_N_P | 9 | 10 | 82 | 28934 | 28953 | N,14 |
| Ch_N_R | 23 | 9 | 2353 | 28979 | 28958 | N |
| Ch_ORF1ab_F | 6 | 6 | 20 | 13342 | 13362 | nsp10 |
| Ch_ORF1ab_P | 20 | 8 | 204 | 13377 | 13404 | nsp10 |
| Ch_ORF1ab_R | 11 | 6 | 222 | 13460 | 13442 | nsp11, nsp12 |
| E_Sarbeco_F | 13 | 8 | 12 | 26269 | 26294 | nsp12 |
| E_Sarbeco_P1 | 26 | 12 | 55 | 26332 | 26357 | E |
| E_Sarbeco_R | 14 | 7 | 60 | 26381 | 26360 | E |
| HKU- ORF1b-nsp14R | 14 | 8 | 118 | 18909 | 18889 | E |
| HKU-NF | 20 | 7 | 625 | 29145 | 29166 | nsp14 |
| HKU-NP | 13 | 8 | 1154 | 29196 | 29177 | N |
| HKU-NR | 24 | 7 | 284 | 29254 | 29236 | N |
| HKU-ORF1b-nsp141P | 10 | 7 | 700 | 18849 | 18872 | N |
| HKU-ORF1b-nsp14F | 12 | 5 | 615 | 18778 | 18797 | nsp14 |
| N_Sarbeco_F | 15 | 8 | 633 | 28706 | 28724 | nsp14 |
| N_Sarbeco_P | 25 | 12 | 295 | 28753 | 28777 | N |
| N_Sarbeco_R | 25 | 10 | 1165 | 28833 | 28814 | N,14 |
| nCoV_2019 Forward | 3 | 6 | 173 | 15216 | 15238 | N,14 |
| nCoV_2019 probe | 4 | 1 | 0 | 15297 | 15280 | nsp12 |
| nCoV_2019 Reverse | 5 | 3 | 7 | 15320 | 15298 | nsp12 |
| nCoV_IP2-12669Fw | 7 | 6 | 28 | 12690 | 12707 | nsp9 |
| nCoV_IP2-12696bProbe(+) | 13 | 8 | 166 | 12717 | 12737 | nsp9 |
| nCoV_IP2-12759Rv | 13 | 9 | 245 | 12797 | 12780 | nsp9 |
| nCoV_IP4-14059Fw | 3 | 4 | 205 | 14080 | 14098 | nsp12 |
| nCoV_IP4-14084Probe(+) | 17 | 7 | 217 | 14105 | 14123 | nsp12 |
| nCoV_IP4-14146Rv | 15 | 9 | 209 | 14186 | 14167 | nsp12 |
| NIID_2019-nCOV_N_F2 | 9 | 9 | 253 | 29125 | 29144 | N |
| NIID_2019-nCOV_N_P2 | 25 | 7 | 2890 | 29222 | 29241 | N |
| NIID_2019-nCOV_N_R2 | 18 | 9 | 268 | 29282 | 29263 | N |
| NIID_WH-1_F24381 | 23 | 11 | 1075 | 24364 | 24384 | S |
| NIID_WH-1_F501 | 20 | 8 | 99 | 484 | 504 | nsp1 |
| NIID_WH-1_F509 | 17 | 6 | 219 | 492 | 510 | nsp1 |
| NIID_WH-1_R24873 | 12 | 9 | 34 | 24856 | 24834 | S |
| NIID_WH-1_R854 | 10 | 7 | 1014 | 837 | 816 | nsp2 |
| NIID_WH-1_R913 | 19 | 10 | 288 | 896 | 874 | nsp2 |
| NIID_WH-1_Seq_F24383 | 22 | 11 | 1075 | 24366 | 24386 | S |
| NIID_WH-1_Seq_F519 | 27 | 6 | 403 | 502 | 521 | nsp1 |
| NIID_WH-1_Seq_R24865 | 10 | 8 | 34 | 24848 | 24830 | S |
| NIID_WH-1_Seq_R840 | 9 | 10 | 210 | 823 | 805 | nsp1 |
| RdRp_SARSr-F | 16 | 5 | 603 | 15431 | 15452 | nsp12 |
| RdRP_SARSr-P1 | 7 | 14 | 1927 | 15469 | 15494 | nsp12 |
| RdRp_SARSr-P2 | 7 | 14 | 1927 | 15470 | 15494 | nsp12 |
| RdRp_SARSr-R | 3 | 7 | 16 | 15530 | 15505 | nsp12 |
| SARS-CoV-2_IBS_E29_F | 18 | 11 | 26 | 26259 | 26280 | E |
| SARS-CoV-2_IBS_E29_R | 10 | 9 | 22 | 26374 | 26356 | E |
| SARS-CoV-2_IBS_m_N 1_F | 10 | 9 | 110 | 29121 | 29140 | N |
| SARS-CoV-2_IBS_m_N 1_R | 22 | 6 | 392 | 29520 | 29501 | N |
| SARS-CoV-2_IBS_m_N 2_F | 24 | 4 | 94 | 28982 | 29001 | N |
| SARS-CoV-2_IBS_m_N 2_R | 26 | 9 | 569 | 29375 | 29356 | N |
| SARS-CoV-2_IBS_m_RdRP 1_F | 7 | 9 | 106 | 15355 | 15374 | nsp12 |
| SARS-CoV-2_IBS_m_RdRP 1_R | 10 | 8 | 412 | 15556 | 15537 | nsp12 |
| SARS-CoV-2_IBS_m_RdRP 2_F | 7 | 4 | 443 | 15167 | 15186 | nsp12 |
| SARS-CoV-2_IBS_m_RdRP 2_R | 8 | 10 | 390 | 15365 | 15346 | nsp12 |
| SARS-CoV-2_IBS_m_S 1_F | 19 | 7 | 39 | 24048 | 24067 | S |
| SARS-CoV-2_IBS_m_S 1_R | 12 | 7 | 7762 | 24338 | 24319 | S |
| SARS-CoV-2_IBS_m_S 2_F | 16 | 8 | 173 | 24137 | 24156 | S |
| SARS-CoV-2_IBS_m_S 2_R | 9 | 5 | 172 | 24436 | 24417 | S |
| SARS-CoV-2_IBS_N19_F | 14 | 12 | 116 | 28732 | 28751 | N |
| SARS-CoV-2_IBS_N19_R | 25 | 10 | 979 | 28849 | 28830 | N,14 |
| SARS-CoV-2_IBS_RdRP29_F | 6 | 9 | 71 | 15092 | 15111 | nsp12 |
| SARS-CoV-2_IBS_RdRP29_R | 13 | 6 | 443 | 15193 | 15174 | nsp12 |
| SARS-CoV-2_IBS_S29_F | 20 | 6 | 712 | 22340 | 22359 | S |
| SARS-CoV-2_IBS_S29_R | 7 | 12 | 719 | 22447 | 22428 | S |
| WuhanCoV-spk1-f | 21 | 12 | 1075 | 24354 | 24377 | S |
| WuhanCoV-spk2-r | 17 | 7 | 29 | 24900 | 24876 | S |
